# Identification of a Novel Interaction between *Theileria* Prohibitin (*Ta*PHB-1) and Bovine RuvB-Like AAA ATPase 1

**DOI:** 10.1128/spectrum.02502-22

**Published:** 2023-01-18

**Authors:** Prasanna Babu Araveti, Prajna Parimita Kar, Akshay Kuriakose, Achintya Sanju, Kota Arun Kumar, Anand Srivastava

**Affiliations:** a National Institute of Animal Biotechnology (NIAB), Hyderabad, Telangana, India; b Graduate Studies, Regional Centre for Biotechnology (RCB), Faridabad, Haryana, India; c Department of Animal Biology, School of Life Science, University of Hyderabad, Hyderabad, Telangana, India; d Regional Centre for Biotechnology (RCB), Faridabad, Haryana, India; Weill Cornell Medicine

**Keywords:** *Theileria*, yeast two-hybrid, prohibitin, RUVBL-1, coexpression, coimmunoprecipitation

## Abstract

Theileriosis is a tick-borne disease caused by Theileria annulata, an intracellular parasite that belongs to the phylum Apicomplexa. The infective forms of the parasite to cattle are sporozoites that are introduced into the host when the infected ticks take a blood meal. The sporozoites selectively invade bovine B cells, macrophages, or monocytes, leading to their cellular transformation. The parasite factors involved in the host cell transformation are not well explored. In pursuit of this, we revisited the probable secretome of the parasite and, following a stringent downscaling criterion, have identified *Theileria* prohibitin (*Ta*PHB-1) as one of factors secreted into the host cells. Interestingly, in infected cells, *Ta*PHB-1 localized both on the parasite surface and in the host cytoplasm, and independent approaches such as coimmunoprecipitation, yeast two-hybrid screening (Y2H), and liquid chromatography-tandem mass spectrometry (LC-MS/MS) confirmed RuvB-like AAA ATPase 1 (RUVBL-1) as one of its interacting partners. Further, the *T. annulata* infection does not affect the localization of bovine prohibitin. Mitigating the expression of bovine RUVBL-1 precluded the translocation of *Ta*PHB-1 in the host cell cytoplasm without affecting the host cell viability. Taken together, we report for the first time a unique interaction of *Ta*PHB-1 with bovine RUVBL-1 that is likely needed to cause cancer-like hallmarks during theileriosis.

**IMPORTANCE** Theileria annulata is an apicomplexan parasite that causes tropical theileriosis in cattle. It is the only eukaryotic pathogen able to cause cellular transformation of host cells yielding a cancer-like phenotype. The parasite factors responsible for the transformation of the host cell are largely unknown. This study demonstrates for the first time the partial role of *Theileria* prohibitin (*Ta*PHB-1) in maintaining the transformed state of the host cell and its interaction with RuvB-like AAA ATPase 1 (RUVBL-1) in a *T. annulata*-infected bovine cell line. Interestingly, the knockdown of bovine RUVBL-1 rendered the parasites metabolically inactive, implying that the identified interaction is critical for parasite survival. This study contributes to our understanding the *Theileria*-host interactions and offers scope for novel therapeutic interventions to control theileriosis.

## INTRODUCTION

Tropical theileriosis, or Mediterranean fever, is a tick-borne hemoprotozoan disease caused by an intracellular parasite, Theileria annulata. Tropical theileriosis is geographically distributed in various countries in Central Asia, Southern Europe, North Africa, and the Middle East. *T. annulata* is transmitted by Hyalomma anatolicum. It causes disease in Bos taurus (1). The symptoms of the disease include fever, enlarged peripheral lymph nodes, anemia, decline in milk production, and emaciation. Death may occur after 3 to 4 weeks of infection in untreated animals. The economic impact of tropical theileriosis in India is approximately US$800 million per annum (2). Buparvaquone, a hydroxynaphthoquinone, is effective in treating bovine tropical theileriosis (3). Point mutations in *T. annulata* cytochrome *b* (4) and the alanine-to-proline mutation at position 53 of parasite peptidyl-prolyl isomerase (*Ta*PIN1) (5) have been associated with the gain of resistance to buparvaquone, a front-line drug for the treatment of theileriosis. The complex life cycle of *T. annulata* includes gametogony and sporogony in ticks, while merogony and piroplasm stages occur in cattle. The cattle are infected by the inoculation of sporozoites by the tick during the feeding of the blood meal. The sporozoites invade leucocytes (B cells and monocytes) and develop into macroschizonts. The macroschizonts differentiate into microschizonts and ultimately into the merozoites that infect erythrocytes, in which they develop into piroplasm. The piroplasms are taken up by the tick, and they undergo gametogony in the gut epithelial cells of the tick and finally develop into sporozoites by sporogony in the salivary glands.

The macroschizont is one of the most important stages that can subvert various signaling pathways leading to the transformation of the host leucocytes (6, 7). The secreted parasite proteins are likely the effectors in this process, as killing the parasite leads to the death of the host cells (8). Several lines of evidence point to the role of host and parasite protein interactions in the transformation of the host cell. For example, the parasite peptidyl-prolyl isomerase (*Ta*PIN1) is secreted into host cytosol and interacts with host ubiquitin ligase FBW7, leading to its degradation and subsequent stabilization of c-JUN, promoting transformation (9). *T. annulata* p104 protein (*Ta*-p104) along with a microtubule and SH3 domain-interacting protein (*Ta*MISHIP) interacts with the host adaptor protein CD2AP, which is believed to play a crucial role in cytokinesis, as the overexpression of *Ta*MISHIP in noninfected bovine macrophages leads to binucleation (10). Also, the secreted *T. annulata Ta*9 protein contributes to the activation of the host AP-1 transcription factor and contributes to leukocyte transformation (11). Some other host-parasite interactions are reported that involve the host cell transformation but are not obligatory for survival of the parasite within the host. *T. annulata* cysteine proteinase interacts with two host proteins, cereblon transcript variant X2 and protein phosphatase 4 catalytic subunit, which are involved in cellular processes such as microtubule organization, DNA repair, and cell apoptosis (12). *T. annulata* cyclophilin 1 interacts with host cell MED21, which is usually involved in regulating the transcription of RNA polymerase II-dependent genes, but knockdown of MED21 in *T. annulata*-infected leucocytes did not affect NF-κB signaling (13). *T. annulata* surface protein (*Ta*SP) interacts with host microtubulin (14). During host cell mitosis, *Ta*SP colocalizes and interacts with the spindle poles, which suggests that this interaction has a potential role in parasite distribution into the host cells (14). Also, *Ta*SP is phosphorylated by the host cell kinase CDK1, which plays a crucial role in cell division (15).

Prohibitins are highly conserved proteins found in all eukaryotes. They belong to the stomatin/prohibitin/flotillin/HfIK/C (SPFH) family. They play a significant role in transcription, nuclear signaling, mitochondrial structural integrity, cell division, and cell membrane metabolism (16). Distinct functions of prohibitin protein have been observed in various parasites. For example, prohibitin in Trypanosoma brucei is located in the mitochondrion, where it maintains mitochondrial membrane potential (17). Prohibitin in Leishmania donovani interacts with the host HSP70 present on the macrophage surface, which may help the parasite to escape invasion by host macrophages (18). In the case of Plasmodium berghei, prohibitin regulates mitochondrial membrane polarity, and prohibitin-deficient parasites cause mitochondrial depolarization in the mosquito vector, which in turn, leads to a block in transmission (19). However, the role of prohibitin in the *T. annulata* parasite is unknown. Previously, it was speculated that prohibitin might have a role in the transformation of the host cell (20). In this study, we characterized the prohibitin of *T. annulata* and identified bovine RuvB-like AAA ATPase 1 (RUVBL-1) as one of the host proteins with which it interacts. We show for the first time that the host cellular levels of RUVBL-1 regulate the viability of intracellular *Theileria* parasites, and the growth and secretory activities of the parasite are compromised when host RUVBL-1 levels are diminished.

## RESULTS

### Prohibitins are highly conserved proteins.

**FIG 1:**
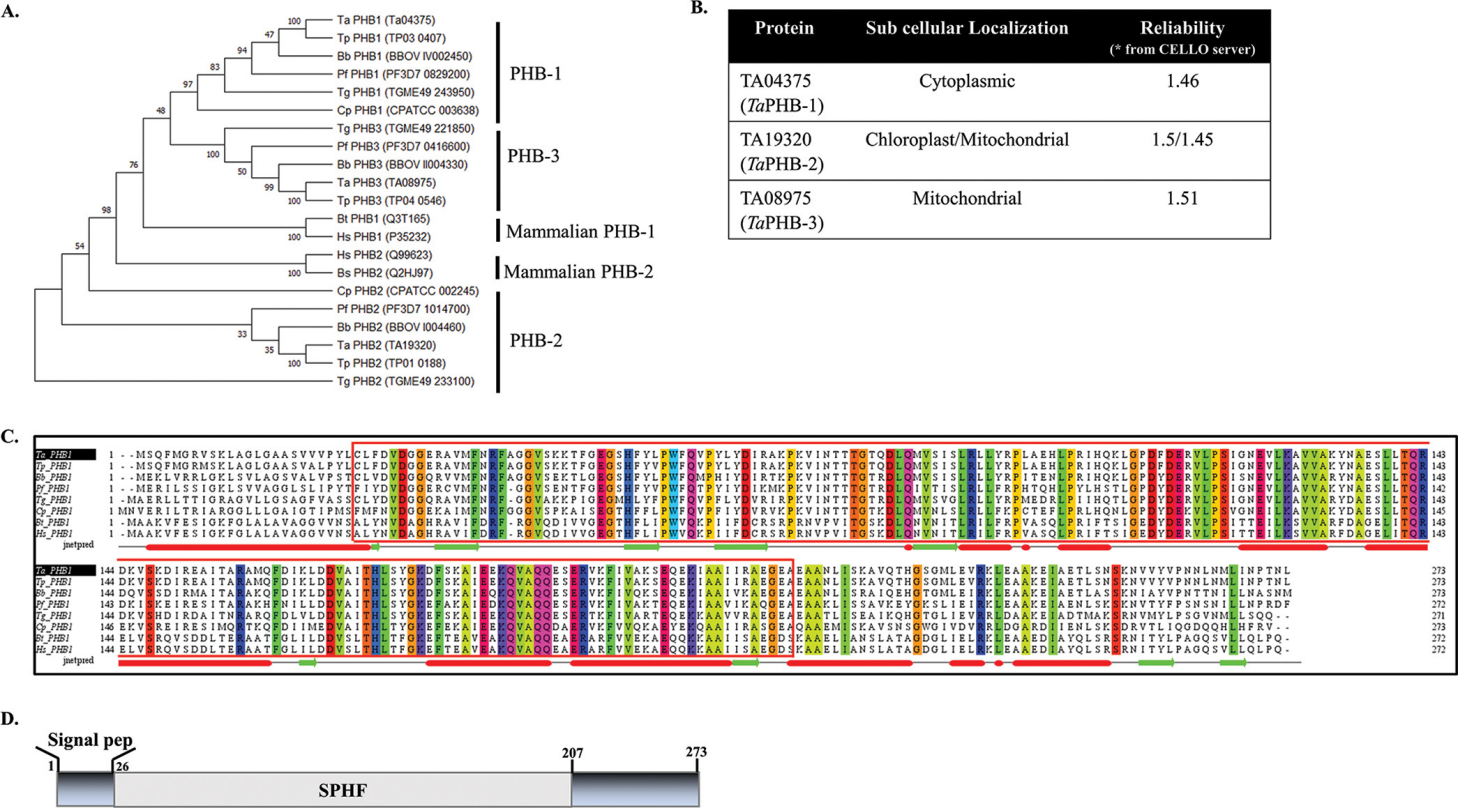
*In silico* analysis of prohibitins of *T. annulata*. (A) Phylogenetic tree showing a closer evolutionary relatedness of *T. annulata* prohibitins (*Ta*PHB-1, *Ta*PHB-2, and *Ta*PHB-3) to *Theileria parva* and Babesia bovis. *Ta*PHB-1 and *Ta*PHB-2 are distantly related to Cryptosporidium parvum. *Ta*PHB-3 is absent in Cryptosporidium parvum and in higher eukaryotes such as Bos taurus and Homo sapiens, while *Ta*PHB-1 and *Ta*PHB-2 are distantly related to PHB-2 of Bos taurus and Homo sapiens. (B) Subcellular localization prediction of *T. annulata* prohibitins using CELLO. The scores indicated in the table envisage cytoplasmic localization for *Ta*PHB-1 (TA04375) and a mitochondrial localization for *Ta*PHB-2 (TA19320) and *Ta*PHB-3 (TA08975). (C) Multiple-sequence alignment of *Ta*PHB-1 (TA04375) with PHB-1 of other apicomplexan parasites. *Theileria parvum* (Tp PHB-1), Babesia bovis (Bb PHB-1), Plasmodium falciparum (Pf PHB-1), Toxoplasma gondii (Tg PHB-1), Cryptosporidium parvum (Cp PHB-1), Bos taurus (Bt PHB-1), and Homo sapiens (Hs PHB-1). The SPFH domain is highlighted by the red box. The conserved amino acid residues across all prohibitins are color coded. The secondary structure elements of *Ta*PHB-1 are shown below the sequences: red represents α helices, green represents β strands, and gray represents random coils. (D) Schematic showing the domain organization of *Ta*PHB-1. Amino acids 1 to 26 and 27 to 207 encompass the signal peptide and SPFH domain, respectively.

Using SignalP 3.0, we screened the entire proteome of *T. annulata* to identify proteins bearing a signal peptide. Our search led to the identification of a total of 438 proteins (see Sheet S1 in the supplemental material). Of these 438 proteins, 49 were found to be expressed in the schizont stage based on the available experimental *T. annulata* schizont proteome data (21, 22). Of the 49 proteins, 25 were nonhypothetical or annotated (Fig. S1 and Sheet S1), which is consistent with the previously published data (20). We selected prohibitin(s) from these 25 proteins for further analysis. The phylogenetic analysis suggests that the apicomplexan prohibitins branch away from the higher eukaryotes such as Bos taurus and Homo sapiens (Fig. 1A), which is also evident in the percentage identity matrix (Fig. S2A). Even among the apicomplexans, the *T. annulata* prohibitins are closely related to Babesia bovis and distantly related to Cryptosporidium parvum. We also observed the presence of an additional prohibitin (PHB-3) in apicomplexan parasites except in Cryptosporidium parvum. Based on the phylogenetic analysis, three putative prohibitin-like proteins of *T. annulata* were named TA04375 (*Ta*PHB-1), TA19320 (*Ta*PHB-2), and TA08975 (*Ta*PHB-3). The multiple-sequence alignment and the identity matrix of the three *T. annulata* prohibitins suggested that *Ta*PHB-1 is the most divergent (Fig. S2B and S2C). The prediction for subcellular localization of the three *T. annulata* putative prohibitins was performed using the CELLO server, which indicated likely cytoplasmic localization of *Ta*PHB-1 and a mitochondria/chloroplast localization for the other two prohibitins. (Fig. 1B). Based on these predictions, we hypothesize a possible translocation of *Ta*PHB-1 to the host cell cytoplasm and a possibility of interacting with the host protein(s), contributing to cellular transformation. Hence, we selected *Ta*PHB-1 for further investigations.

Multiple-sequence alignment of amino acid sequences of prohibitin-1 (PHB-1) from apicomplexan parasites (*Theileria annulata*, Theileria parva, Plasmodium falciparum, Toxoplasma gondii, Cryptosporidium parvum, Babesia bovis), human (Homo sapiens), and bovine (Bos taurus) indicated that these proteins are highly conserved and possess the SPFH domain with a consensus sequence KQVAQQ (Fig. 1C). The domain analysis of *Ta*PHB-1 is shown in Fig. 1D.

### Affinity-purified *Ta*PHB-1 antibodies are specific to Ana2014 cells.

**FIG 2:**
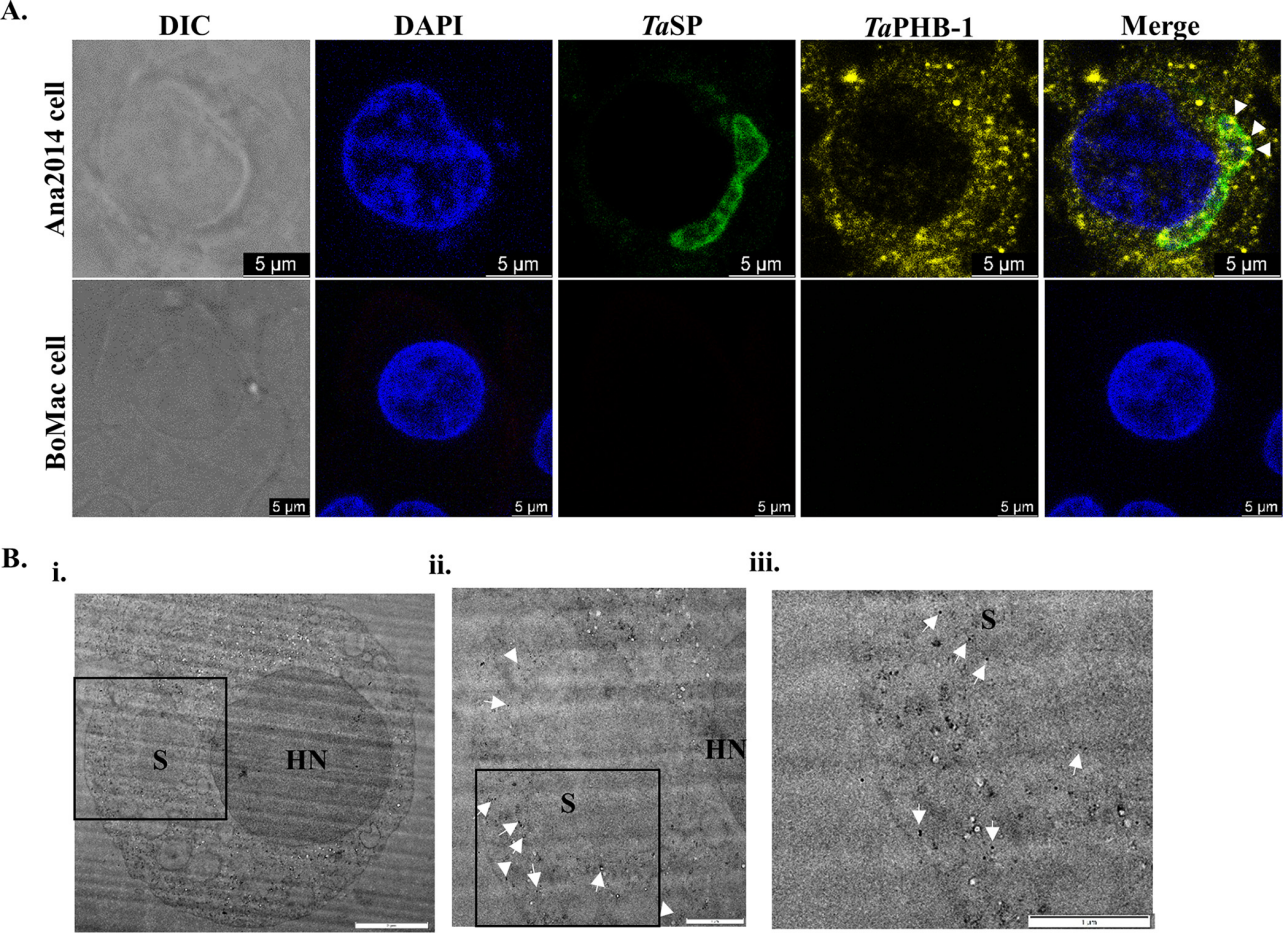
Subcellular localization of *Ta*PHB-1 in Ana2014 cells. (A) Indirect immunofluorescence assay (IFA) for localizing *Theileria* and secreted *Ta*PHB-1 in Ana2014 (top panel) and BoMac cells (bottom panel). In the top panel, the *Theileria annulata* parasite is stained with chicken anti-*Ta*SP antibody, and immunoreactivity was revealed with anti-chicken secondary antibody conjugated to Alexa Fluor 488. The secreted *Ta*PHB-1 was detected with a mouse polyclonal antibody and revealed with an anti-mouse secondary antibody conjugated to Alexa Fluor 555. In the lower panel, no immunoreactivity was detected for *Ta*SP or *Ta*PHB-1, suggesting no cross-reactivity with the host cell. DAPI was used to stain host and parasite nuclei in both panels. Scale bar = 5 μm. (B) Immuno-electron microscope image of Ana2014 cell labeled for *Ta*PHB-1. The arrows show the 10- nm gold nanoparticles on the parasite surface and host cytosol. S, schizont; HN, host nucleus. Scale bar = (i) 2 μm, (ii) 1 μm, and (iii) 1 μm (zoomed).

*T. annulata* surface protein (*Ta*SP) is widely used as a cell surface marker for *T. annulata* schizonts (23). Thus, we recombinantly expressed and purified *Ta*SP. The *Ta*SP protein is known to have an abnormal separation on SDS-PAGE (23). The expected molecular weight of recombinant *Ta*SP is 15.4 kDa, while we observed that it migrates at 36 kDa on SDS-PAGE (Fig. S3). The antibodies raised in chicken against *Ta*SP recognized native protein in the parasite lysate and stained the parasite cell surface (Fig. S3 and Fig. 2A).

Our efforts to express *Ta*PHB-1 as a soluble protein remained unsuccessful. Thus, we purified recombinant His-tagged *Ta*PHB-1 under denatured conditions (Fig. S4A). Prior to immunization, we confirmed by liquid chromatography-tandem mass spectrometry (LC-MS/MS) that the recombinantly expressed protein was indeed *Ta*PHB-1 (Fig. S5). Mouse antiserum was generated against denatured *Ta*PHB-1 and affinity-purified against recombinant His-tagged *Ta*PHB-1 (Fig. S4A). The anti-*Ta*PHB-1 antibody detected recombinant His-tagged *Ta*PHB-1 in Western blot analysis (Fig. S4A) and showed an expected size of 30 kDa. In the Western blot analysis, the affinity-purified antibodies against *Ta*PHB-1 recognized *Ta*PHB-1 protein at a higher molecular size (~60 kDa) in the lysates of Ana2014 cells but not from bovine macrophage cell line (BoMac) lysate (Fig. S4B), suggesting the specificity of affinity-purified *Ta*PHB-1 antibodies.

### *Ta*PHB-1 is exported to the host cytoplasm.

We next analyzed if *Ta*PHB-1 was exported into the host cytoplasm based on our previous observation that *Ta*PHB-1 has a signal peptide. We performed an indirect immunofluorescence assay (IFA) with affinity-purified anti-*Ta*PHB-1 antibodies to analyze the cellular distribution in infected and uninfected cells (Fig. 2A). Anti-*Ta*SP antibody was used for labeling the parasite surface, and DAPI (4′,6-diamidino-2-phenylindole) was used to stain host and parasite nuclei. Interestingly, we noted that the *Ta*PHB-1 localized on the parasite surface as well as in the host cell cytosol (Fig. 2A, top panel). The lack of IFA signals from BoMac cells (Fig. 2A, bottom panel) indicated the specificity of anti-*Ta*PHB-1 antibody only for parasite and not host prohibitin. Further, it was observed that the *Ta*PHB-1 was localized to the parasite surface and host cytosol by immuno-electron microscopy (Fig. 2B). We conclude that *Ta*PHB-1 has a functional signal peptide that allows both its targeting to the parasite surface and secretion into the host cell. Due to technical limitations associated with the gene-targeting approaches in *Theileria* parasites, we were unable to mutate the signal peptide sequence of *Ta*PHB-1 and demonstrate its effect on host cell transformation.

### The localization of bovine prohibitin remains unchanged upon *T. annulata* infection.

**FIG 3:**
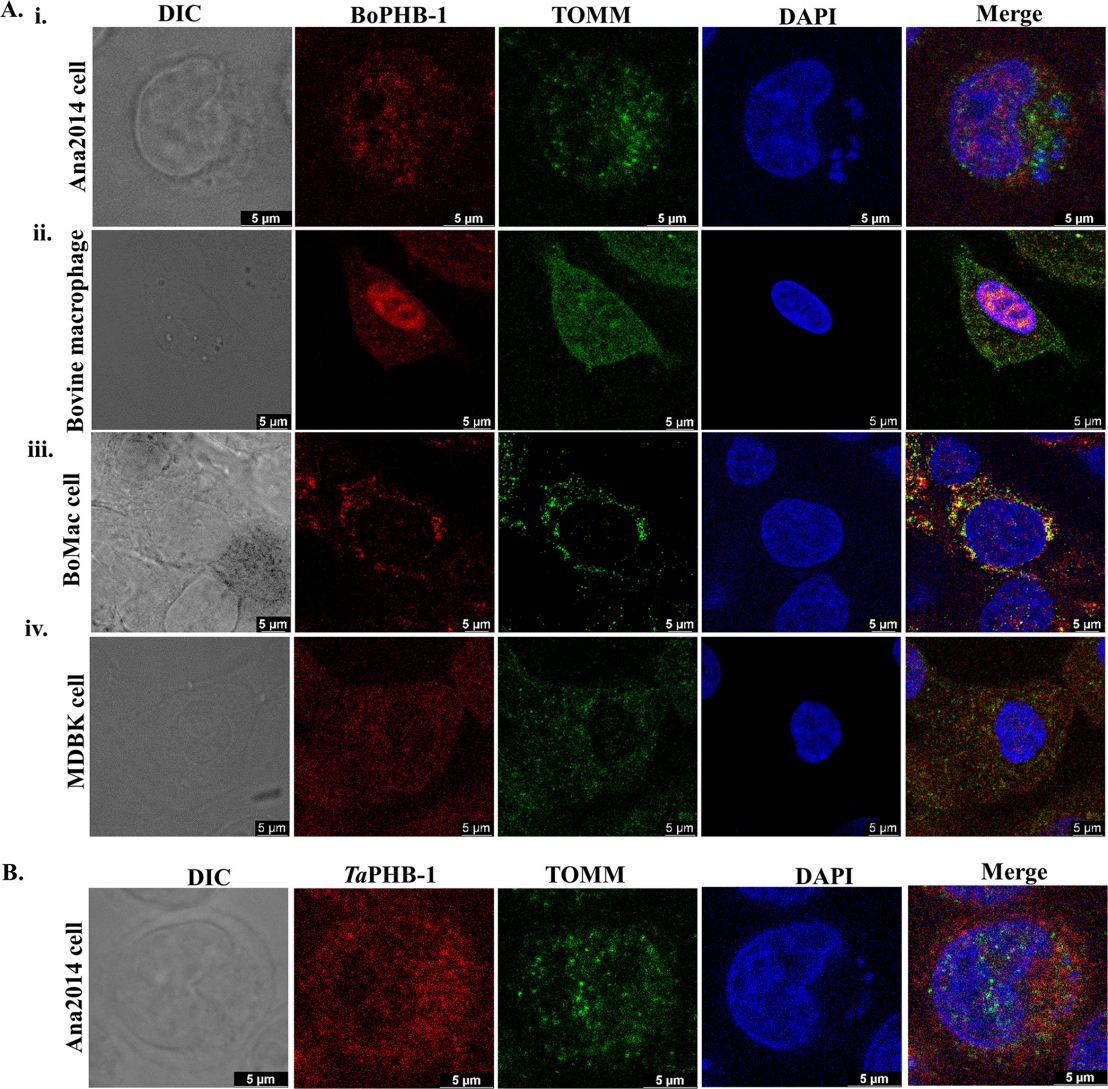
Subcellular localization of bovine PHB-1. (A) Indirect immunofluorescence assay (IFA) for localizing bovine PHB-1: (i) Ana2014 cell, (ii) bovine macrophage cell, (iii) BoMac cell, and (iv) MDBK cell. All cell lines were stained with mouse anti-PHB-1 antibody and revealed with anti-mouse secondary antibody conjugated to Alexa Fluor 647. The mitochondria were visualized using a rabbit anti-TOMM antibody, and immunoreactivity was revealed using an anti-rabbit secondary antibody conjugated to Alexa Fluor 488. Host and parasite nuclei were stained with DAPI. Scale bar = 5 μm. (B) The subcellular localization of *Ta*PHB-1 differs from that of bovine PHB-1 in Ana2014 cells. Ana2014 cells were stained with mouse anti-PHB-1 antibody and revealed with anti-mouse secondary antibody conjugated to Alexa Fluor 647 for localizing *Ta*PHB-1. In Ana2014 cells, the *Ta*PHB-1 was not localized to the mitochondria. Scale bar = 5 μm.

We used commercial antibodies against PHB-1 protein (anti-PHB-1) for the localization of host PHB-1. First, we analyzed the suitability of the anti-PHB-1 to distinguish host PHB-1 and *Ta*PHB-1. We noted that the anti-PHB-1 recognized host PHB-1 as a band at 30 kDa on a Western blot made from lysates of both BoMac cells and Ana 2014 cells (Fig. S6). To analyze the impact of *T. annulata* infection on the localization of bovine prohibitin, we stained both infected and uninfected cells with anti-PHB-1, while the mitochondria were stained with anti-translocase of the outer membrane (TOMM) antibodies. We observed that bovine PHB-1 (BoPHB-1) was significantly localized to the host cell nucleus in Ana2014 cells (Fig. 3Ai) and did not colocalize with TOMM, suggesting its lack of mitochondrial residence in Ana2014 cells (Fig. 3Ai). The same observation was noticed in the uninfected macrophages differentiated from the bovine peripheral blood mononuclear cells (PBMC) (Fig. 3Aii), while in other bovine cell lines (BoMac and Madin-Darby bovine kidney [MDBK]), the BoPHB-1 was observed in the mitochondria (Fig. 3Aiii and 3Aiv). Further, we noted that the exported *Ta*PHB-1 was predominantly in the host cytosol and did not localize to mitochondria, as revealed by TOMM staining (Fig. 3B).

**FIG 4:**
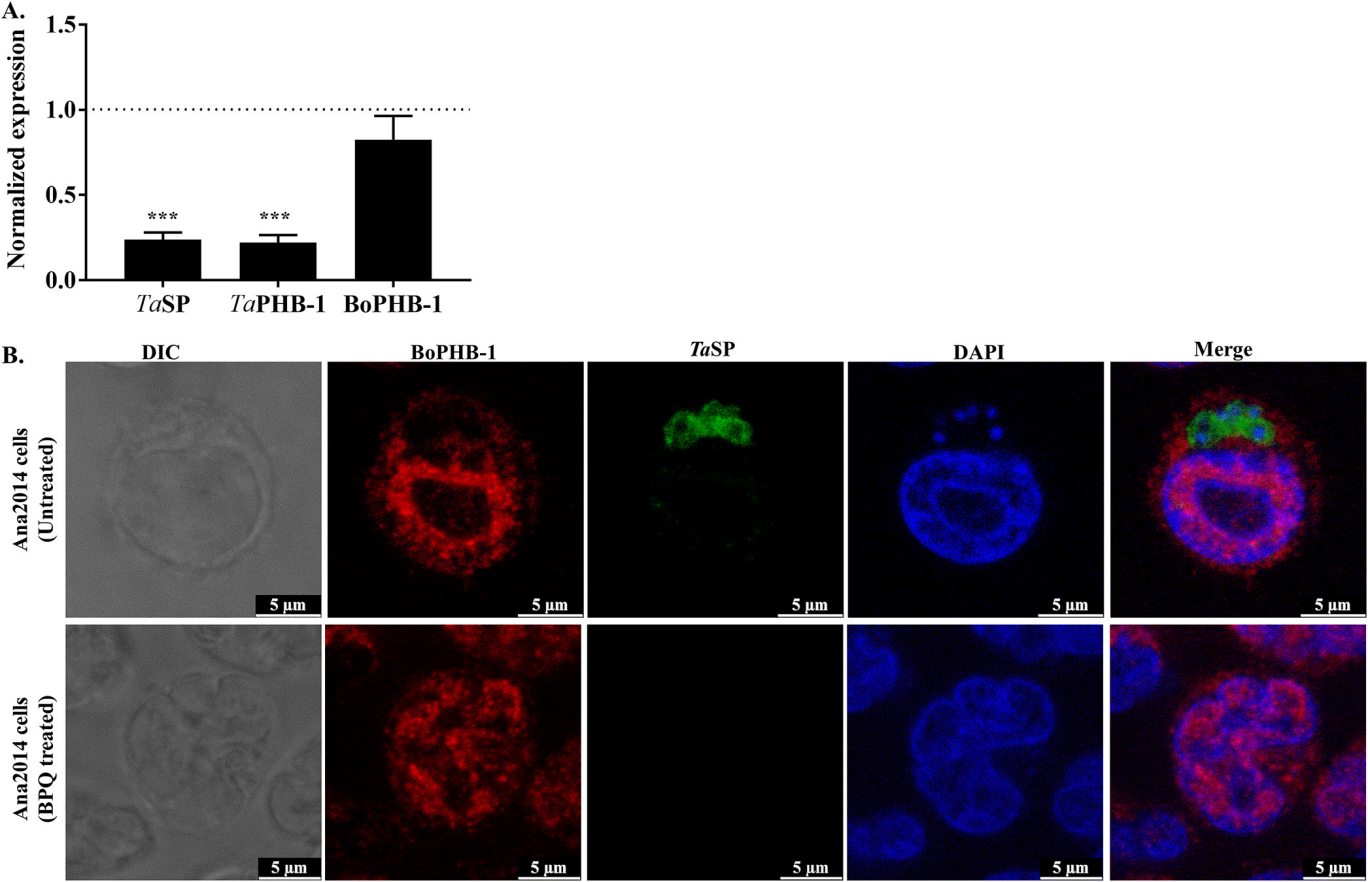
Effect of buparvaquone treatment on the localization of bovine prohibitin in *Theileria annulata*-infected bovine leucocytes. (A) Quantitative real-time PCR showing decreased expression of *Ta*SP and *Ta*PHB-1 transcripts upon buparvaquone treatment, while no significant change was observed for the bovine PHB-1 transcripts. The *y* axis shows normalized expression compared to untreated cultures. (B) Buparvaquone treatment does not affect the cellular distribution of BoPHB-1 in Ana2014 cells. The top panel shows untreated cells, and the bottom panel shows treated cells. Subcellular localization of BoPHB-1 was analyzed using mouse anti-BoPHB-1 antibody and revealed with anti-mouse secondary antibody conjugated to Alexa Fluor 647. *Theileria annulata* parasites were localized using chicken anti-*Ta*SP antibody and revealed with anti-chicken secondary antibody conjugated to Alexa Fluor 488. Host and parasite nuclei were stained with DAPI. The localization of BoPHB-1 was unaffected in Ana2014 cells upon the elimination of the parasite using buparvaquone. Scale bar = 5 μm.

We also assessed the localization of BoPHB-1 in Ana2014 cells upon killing the intracellular parasite. To induce this condition, Ana2014 cells were treated for 72 h with buparvaquone, a theilericidal drug. We observed inhibition of 70% cell proliferation after the treatment (data not shown). The decrease in the gene expression of *Ta*SP confirmed the diminished parasite load upon buparvaquone treatment (Fig. 4A and B). Also, the gene expression levels of *Ta*PHB-1 and BoPHB-1 were reduced (Fig. 4A). BoPHB-1 still localized to the nucleus, as judged by IFA studies, under conditions of reduced parasite load (Fig. 4B). Together, these observations suggest that *T. annulata* infection does not affect the localization of bovine prohibitin.

### The yeast two-hybrid cDNA library (prey library) of Ana2014 cells has a broad coverage of cellular transcripts representing a large number of expressed sequence tags (ESTs).

We prepared a yeast two-hybrid library containing cDNA from the *T. annulata*-infected bovine leucocytes (Ana2014 cells) to identify the protein(s) that could interact with *Ta*PHB-1. The cDNA library ranged between 200 bp and 2,500 bp (Fig. S7), and the titer obtained with pGADT7-Rec in the Y187 yeast strain was 2.4 × 10^6^ CFU. These numbers indicated a reasonably good representation of a large number of independent EST clones that were used for yeast two-hybrid screening.

### The bait protein (*Ta*PHB-1) does not auto-activate reporter genes of Y2HGold yeast cells and is nontoxic.

**FIG 5:**
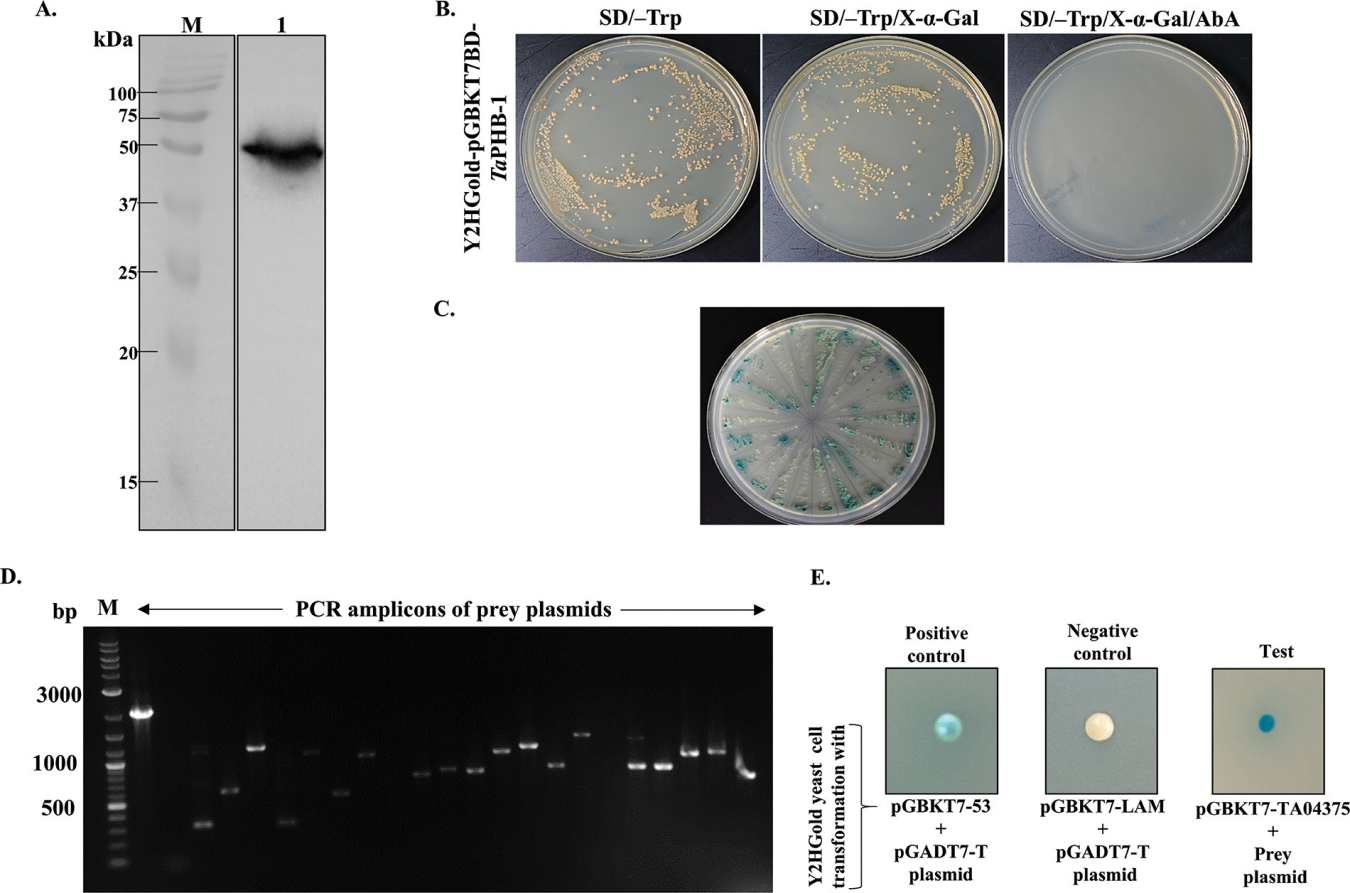
Yeast two-hybrid library screening. (A) Western blot showing the expression of myc-tagged *Ta*PHB-1 in Y2HGold yeast cells. (B) The presence of white colonies on SD/-Trp/X-α-Gal agar plate and no colonies on SD/-Trp/X-α-Gal/AbA plates confirmed the absence of the auto-activation of reporter genes by *Ta*PHB-1 in Y2HGold yeast cells. (C) Plates showing Y2HGold cells that grew on QDO/X/A agar plates after library screening. (D) Agarose gel electrophoresis analysis of PCR products of prey plasmids isolated from the colonies that grew on QDO/X/A agar plates. M, marker. (E) Growth of Y2HGold yeast cells on QDO/X/A agar plates after cotransformation of potential prey plasmid and pGBKT7-*Ta*PHB-1 confirmed their interaction. Cotransformation with pGBKT7-53 and pGADT7-T plasmids was used as a positive control, and cotransformation with pGBKT7-LAM and pGADT7-T plasmids was used as a negative control on the DDO/X/agar plate.

Before mating the prey and bait plasmids in the yeast cells, we ruled out the possibility of auto-activation and toxicity of bait plasmids in the Y2HGold yeast cells. To this end, we cloned the coding sequence of *Ta*PHB-1 in the pGBKT7 plasmid, and its expression was analyzed on a Western blot using an anti-myc antibody. Detection of a myc-tagged *Ta*PHB-1 in the range of 50.3 kDa in Y2HGold yeast cells confirmed the expression in the yeast cells (Fig. 5A). Further, our studies showed that the transformed Y2HGold yeast strain did not exhibit auto-activation of the reporter gene, and *Ta*PHB-1 had no toxic effect on Y2HGold yeast cells (Fig. 5B).

### Yeast two-hybrid library screening led to the identification of bovine RUVBL-1 as an interacting partner of *Ta*PHB-1.

The yeast two-hybrid library screening was performed as described in Materials and Methods. A total of 60 yeast colonies grew after mating and selection of Y2HGold yeast cells containing *Ta*PHB-1 and cDNA library in the Y187 yeast cells on SD/–Leu/–Trp/X-a-Gal/AbA (DDO/X/A) agar plates. All these 60 colonies were patched on highly stringent SD/–Ade/–His/–Leu/–Trp/X-a-Gal/AbAQDO/X/A) agar plates. Only 28 blue colonies out of these 60 patched colonies grew on the QDO/X/A agar plates (Fig. 5C). PCR from each colony led to the identification of various duplicate clones (Fig. 5D). The insert size distribution in the prey plasmid is listed in the Fig. S8. The prey plasmids with unique molecular sizes (PCR product) were rescued with an ampicillin resistance marker in Escherichia coli DH5α cells. All rescued prey plasmids were individually retransformed along with pGBKT7-*Ta*PHB-1 into Y2HGold yeast cells and selected on QDO/X/A agar plates to rule out false positives in previous steps. Only one prey plasmid out of all the plasmids grew on the QDO/X/A agar plate after cotransformation (Fig. 5E). The sequence analysis of confirmed prey plasmid using the NCBI-BLAST tool led to the identification of the interacting gene as Bos taurus RuvB-like AAA ATPase 1 (RUVBL-1).

### *Ta*PHB-1 interacts with bovine RUVBL-1 *in vitro*.

**FIG 6:**
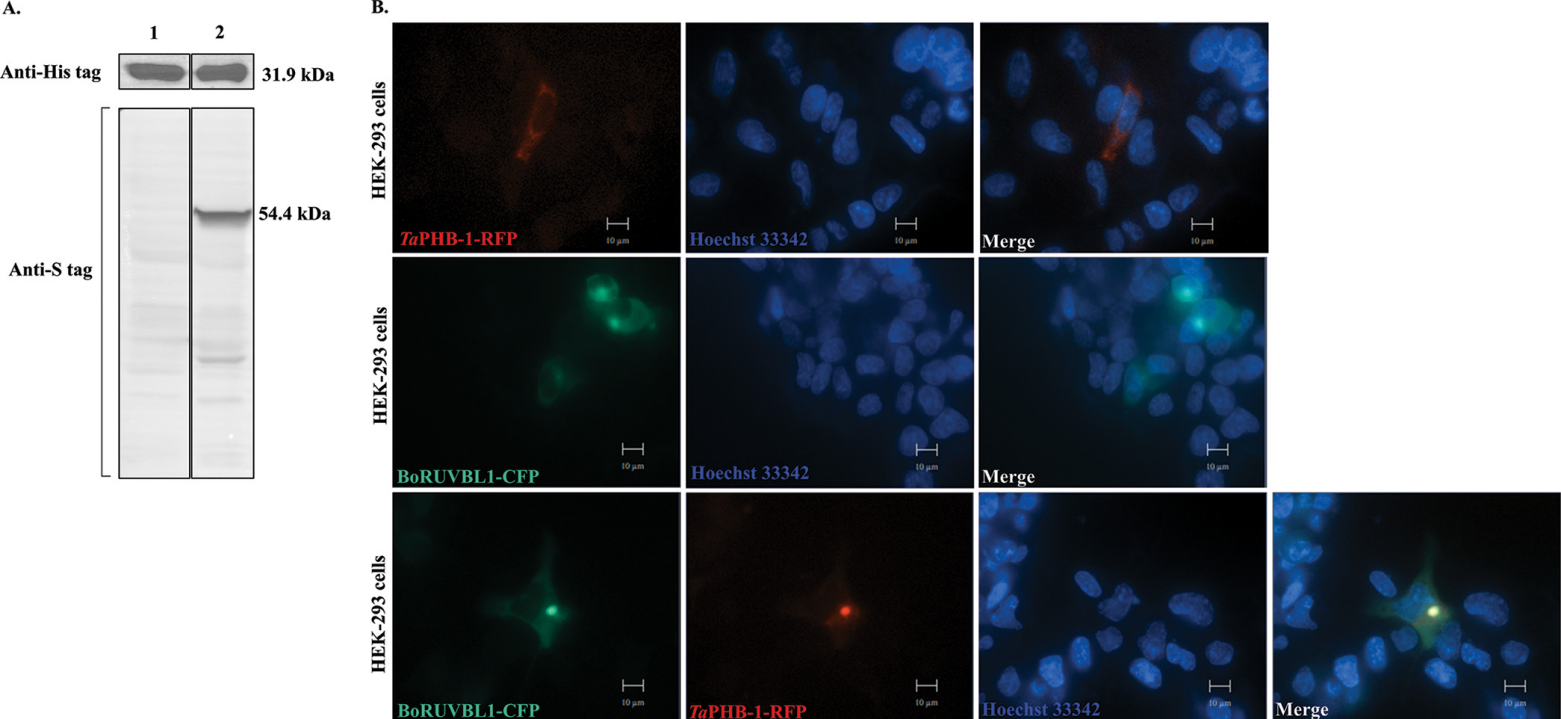
*Ta*PHB-1 interacts with bovine RUVBL-1 *in vitro*. (A) Western blot analysis of *Ta*PHB-1 (6×His tag) and RUVBL-1 (S tag) after pulldown using Ni-NTA beads. (1) Eluate of LemoDE3-pETDuet1-*Ta*PHB-1 bacterial cell lysate after pulldown. (2) Eluate of LemoDE3-pETDuet1-*Ta*PHB-1-RUVBL-1 bacterial cell lysate after pulldown. (B) Colocalization of *Ta*PHB-1 (RFP tag) and bovine RUVBL-1 (CFP-tag) in HEK293 cells. HEK293 cells were transfected with pcDNA3RFP-*Ta*PHB-1 (top panel) or pcDNA3CFP-BoRUVBL1 (middle panel), or cotransfected with both the plasmids (bottom panel) using Lipofectamine 3000 reagent. The fluorescence microscopy images were captured at 24 h posttransfection. HEK293 cell nuclei were stained with Hoechst 33342. Scale bar = 10 μm.

The interaction of *Ta*PHB-1 and bovine RUVBL-1 (Bo-RUVBL-1) was reconfirmed by coexpression *in vitro*. We used pETDuet-1 vector to coexpress both the proteins in E. coli Lemo21(DE3) competent cells. The *Ta*PHB-1 was cloned into multiple-cloning site 1 (MCS1) of pETDuet-1, and full-length *B. taurus* RUVBL-1 was cloned into MCS2 of pETDuet-1. Both proteins were expressed in the E. coli Lemo21(DE3) cells (Fig. S9). After induction with IPTG (isopropyl-β-d-thiogalactopyranoside), His-tagged *Ta*PHB-1 was purified. The detection of S-tagged Bo-RUVBL-1 in the His-tagged pulldown of *Ta*PHB-1 further validated the interaction between the two (Fig. 6A). We also independently confirmed cellular colocalization of red fluorescent protein (RFP)-tagged *Ta*PHB-1 and cyan fluorescent protein (CFP)-tagged bovine RUVBL-1 in HEK293 cells, further reiterating that *Ta*PHB-1 interacts with Bo-RUVBL-1 (Fig. 6B).

### *Ta*PHB-1 interacts with Bos taurus RUVBL-1 in Ana2014 cells.

**FIG 7:**
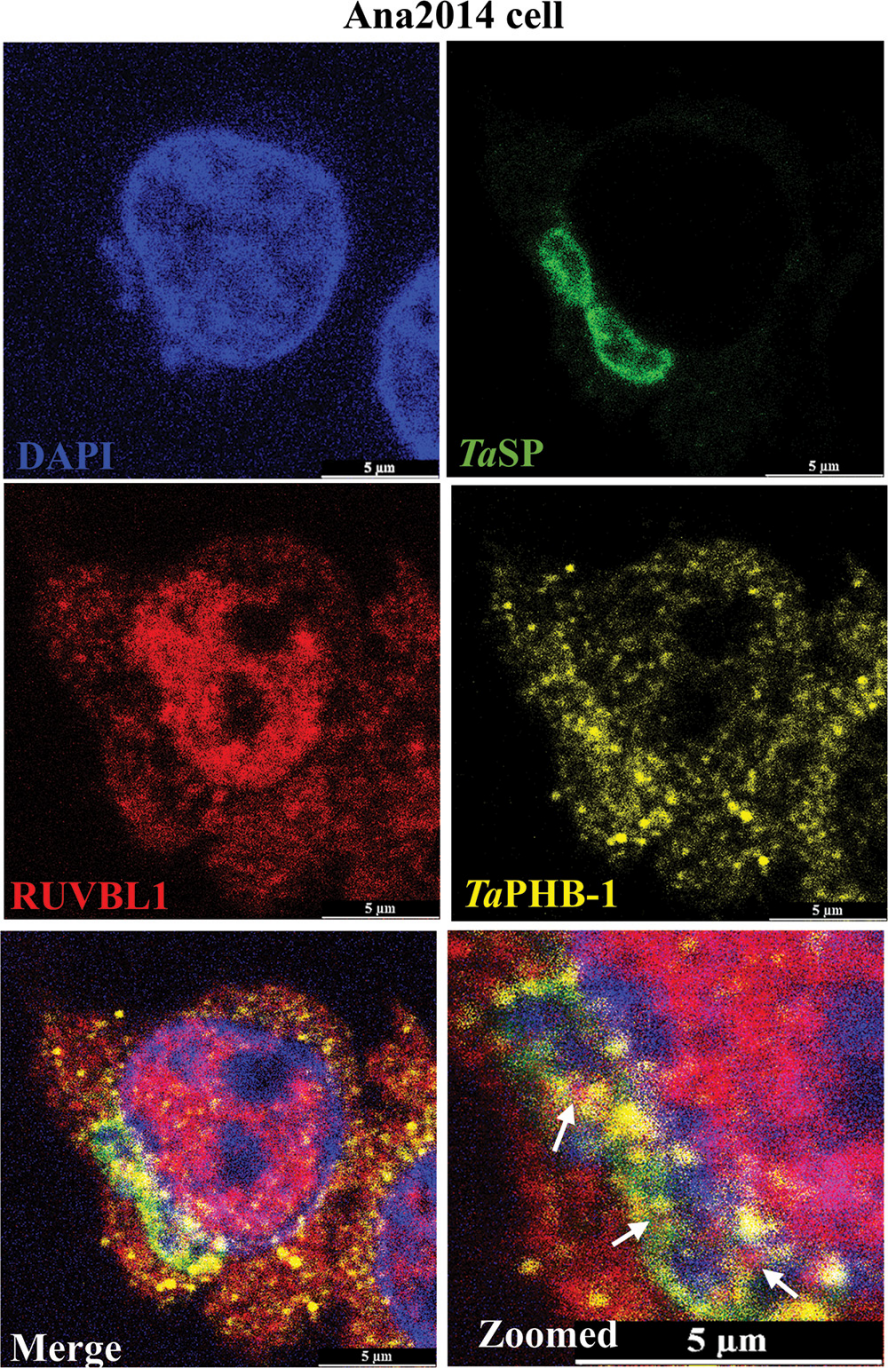
*Ta*PHB-1 colocalizes with bovine RUVBL-1 on the surface of the parasite in Ana2014 cells. Ana2014 cells were immunostained with *Ta*PHB-1, *Ta*SP, and RUVBL-1 antibodies, followed by staining with Vectashield antifade mounting medium with DAPI. The arrows indicate the sites of colocalization of *Ta*PHB-1 and Bo-RUVBL-1 on the parasite surface.

The interaction of *Ta*PHB-1 with Bo-RUVBL-1 in Ana2014 cells was confirmed by colocalization studies using antibodies specific to *Ta*PHB-1 and host RUVBL-1. The bovine RUVBL-1 was mainly localized in the host cell nucleus (Fig. 7 and Fig. S10A). The colocalization of *Ta*PHB-1 and Bo-RUVBL-1 was observed significantly on the parasite surface (Fig. 7), which was labeled using anti-*Ta*SP antibodies.

Further, to analyze the effect of parasite infection on the localization of the Bo-RUVBL-1, we treated Ana2014 cells with buparvaquone. The quantification of Bo-RUVBL-1-specific transcript showed no significant decrease in buparvaquone-treated Ana2014 cells (Fig. S10B). Similarly, there was no significant change in the localization of Bo-RUVBL-1 following the inactivation of the parasite that was evident from a decrease in the expression of parasite proteins, *Ta*SP and *Ta*-PHB-1 (Fig. 4A and Fig. S10C).

**FIG 8:**
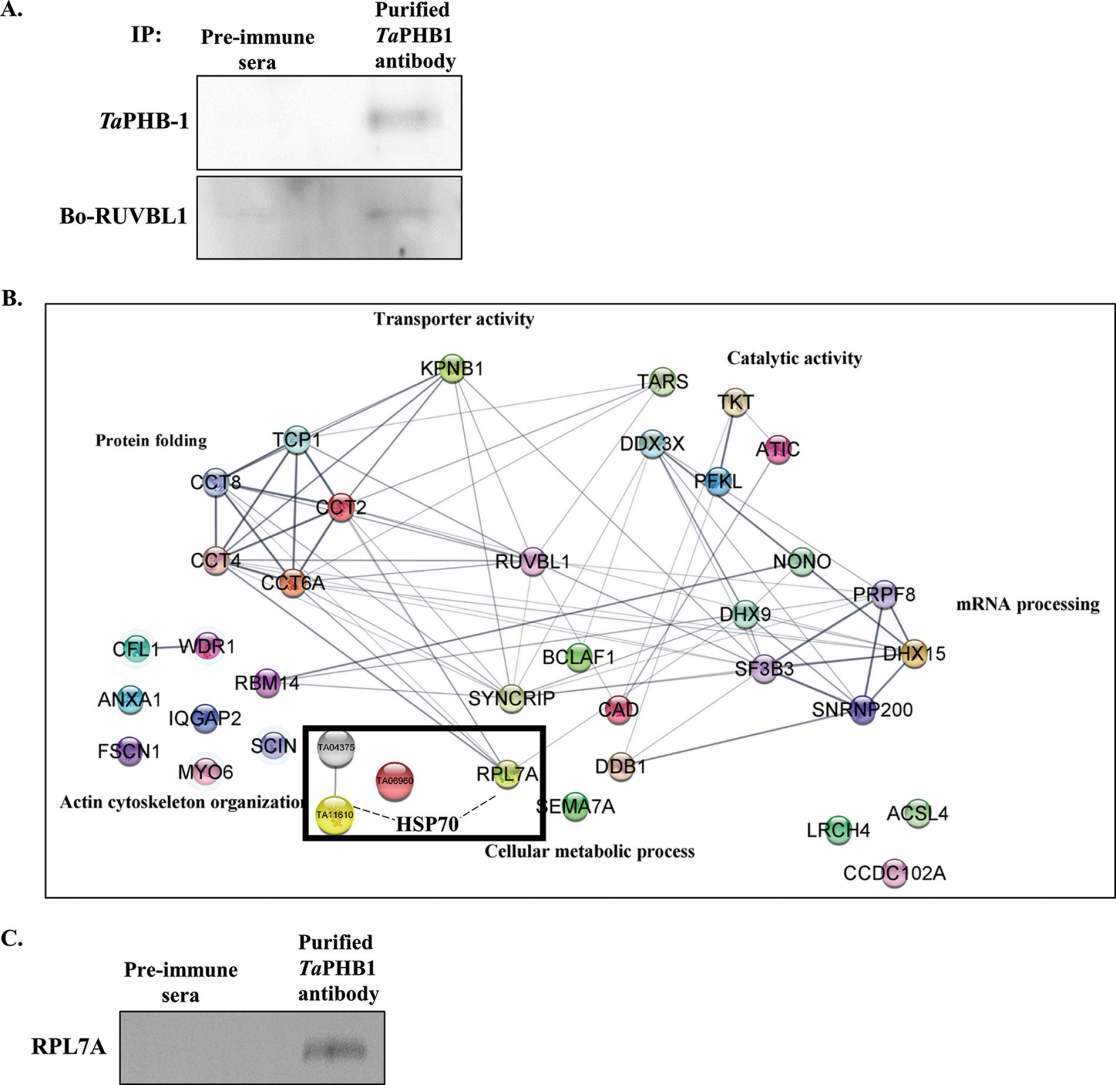
Coimmunoprecipitation of Ana2014 cell lysate using *Ta*PHB-1 affinity-purified antibodies and LC-MS/MS analysis of the interactome. (A) Western blot analysis of eluate of co-IP of Ana2014 cells using affinity-purified *Ta*PHB-1 antibodies. Preimmune serum was used as a control. (B) STRING analysis of the proteins obtained in the *Ta*PHB-1 co-IP experiment (after subtraction with proteins obtained from preimmune serum co-IP). (C) Confirmation of LC-MS/MS data. Western blot analysis of eluate of co-IP of Ana2014 cells using affinity-purified *Ta*PHB-1 antibodies. RPL7A protein identified in the LC-MS/MS analysis was present in the pulldown.

To validate our colocalization studies, we resorted to coimmunoprecipitation studies to show the specific nature of the interaction between *Ta*PHB-1 and Bo-RUVBL-1. Coimmunoprecipitation of Ana2014 cell lysate using affinity-purified *Ta*PHB-1 antibodies followed by the analysis of eluate by Western blotting and LC-MS/MS confirmed the interaction between *Ta*PHB-1 and bovine RUVBL-1 (Fig. 8A and B). Preimmune sera were used as a negative control in coimmunoprecipitation. The LC-MS/MS analysis identified 44 bovine proteins and 2 parasite proteins with more than 10 unique peptides with a less than 0.05 false-discovery rate (Fig. 8B, Sheet S2). We further confirmed the result of LC-MS/MS by Western blot analysis of one of the proteins, 60S ribosomal protein L7a (RPL7A), identified in the mass spectrometry analysis (Fig. 8C). The interactome analysis of *Ta*PHB-1 using STRING suggests that the proteins involved in protein folding, the cellular metabolic process, mRNA processing, catalytic activity, transporter activity, and actin cytoskeleton organization are present in the *Ta*PHB-1 interactome (Fig. 8B).

### Knockdown of bovine RUVBL-1 hinders the translocation of *Ta*PHB-1 to the host cytoplasm.

**FIG 9:**
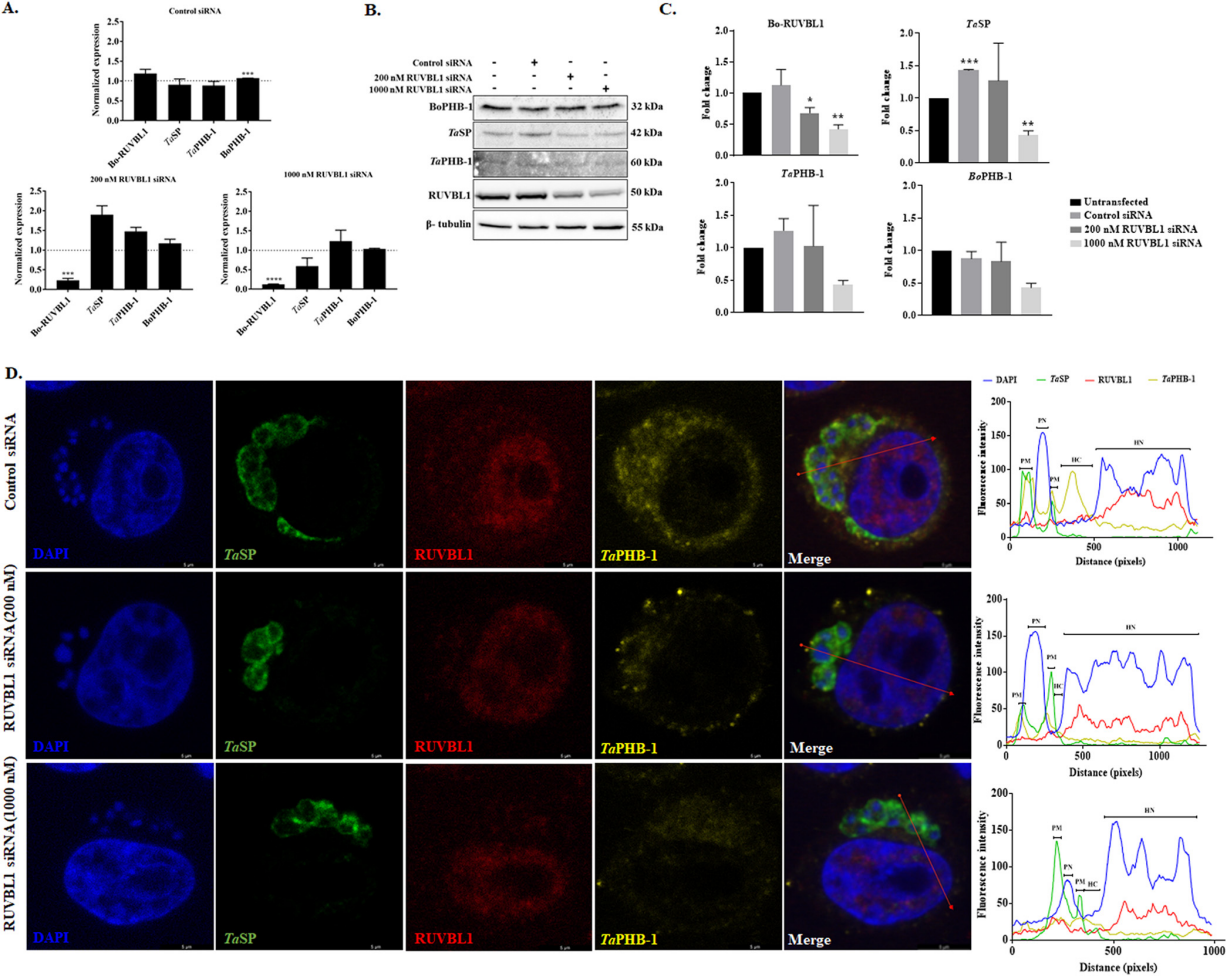
Silencing of bovine RUVBL-1 hinders the localization of *Ta*PHB-1 to the host cytosol in Ana2014 cells. (A) qRT-PCR showing a significant decrease in the mRNA levels of Bo-RUVBL-1 and *Ta*SP upon transfection with siRNA targeting Bo-RUVBL-1. No significant changes in the mRNA levels of *Ta*PHB-1 and BoPHB-1 were noted. (B) Western blots showing the decrease in the protein levels of Bo-RUVBL-1, *Ta*SP, *Ta*PHB-1, and BoPHB-1 upon silencing the Bo-RUVBL-1 using siRNA. (C) Fold change in the protein expression levels of Bo-RUVBL-1, *Ta*SP, *Ta*PHB-1, and BoPHB-1 upon silencing the Bo-RUVBL-1 after densitometric normalization of Western blot data. (D) IFA images showing that the change in localization of *Ta*PHB-1 is limited to the parasite membrane upon silencing the Bo-RUVBL-1 in Ana2014 cells. PM, parasite membrane; PN, parasite nucleus; HC, host cytoplasm; HN, host nucleus. Scale bar = 5 mm.

Three sets of small interfering RNAs (siRNAs) were used to knock down the bovine RUVBL-1 in Ana2014 cells. A significant decrease in the levels of Bo-RUVBL-1 was observed at both the mRNA and protein levels (Fig. 9A to C). Also, there was a decrease in the expression of *Ta*SP at both the mRNA and protein levels. The knockdown of Bo-RUVBL-1 did not affect the viability of the Ana2014 cells as analyzed by the trypan blue dye exclusion method (data not shown). The localization studies showed that upon knockdown of expression of the Bo-RUVBL-1, *Ta*PHB-1 was mostly observed on the parasite surface, and there was a decrease in localization of *Ta*PHB-1 in the host cytoplasm (Fig. 9D). Furthermore, the localization of *Ta*SP and BoPHB-1 remains unchanged (Fig. S10D).

## DISCUSSION

Some species of the *Theileria* parasite, such as *T. annulata* and *T. parva*, have a unique ability to transform the host cells. The *Theileria* sporozoites invade host leucocytes and can control host cell proliferation (6). Cellular transformation does not occur when the intracellular parasite is killed (7), suggesting that parasite factors likely facilitate the cellular takeover. To account for this plausible mechanism of cellular transformation, we reasoned the ability of parasites to introduce effectors into host cells using canonical secretory pathways, and therefore we analyzed all parasite proteins bearing a signal peptide. This approach helped us to shortlist a few potential proteins that concurred with previous studies (9, 20). Among these proteins, we prioritized investigating the function of *Ta*PHB-1 (TA04375), based on the general notion that the mammalian orthologue of this protein is overexpressed in cancer cells.

Prohibitins are evolutionarily conserved proteins implicated in a wide variety of cellular functions. In the current study, a strong prediction score for *Ta*PHB-1 to be cytoplasmic, together with the signal peptide-bearing sequence, led us to prioritize *Ta*PHB-1 for studying its role in host cell interactions.

Analyzing the cellular distribution of *Ta*PHB-1 revealed its association with the parasite surface and in the host cytoplasm of an infected bovine macrophage cell line, but not noninfected cells, reiterating the specificity of the anti-*Ta*PHB-1 antibody. The cellular distribution of mammalian prohibitin may serve as a cue for the regulation of apoptotic signals (24), and during the early stages of apoptosis, PHB-1 translocates to the nucleus from the cytosol (25). In line with this notion, we analyzed the distribution of bovine PHB-1 in transformed and nontransformed host cells. The nuclear localization of PHB-1 in uninfected bovine macrophages is likely indicative of an apoptotic event owing to the mortal nature of these primary cells. Interestingly, a nuclear localization of host PHB-1 was also evident in Ana2014 cells, possibly predisposing these cells to apoptosis. However, concomitant secretion of *Ta*PHB-1 in the host cytosol as noted in our study may be a strategic mechanism to counteract the apoptotic signal. Further experimental validation of these observations is required to prove this hypothesis. Conversely, the mitochondrial localization of PHB-1 in BoMac and MDBK cell lines is consistent with their role in protection from apoptosis (24). Since buparvaquone treatment kills the parasite, it is expected that the parasite fails to export *Ta*PHB-1 into the host cell. The absence of secreted *Ta*PHB-1 in host cells may trigger apoptosis, as evident from the nuclear localization of the host PHB-1 during theilericidal treatment.

Our study provides unequivocal evidence for *T. annulata* prohibitin (*Ta*PHB-1) interaction with bovine RUVBL-1. RUVBL-1 (also known as pontin, TIP49) is an AAA+ ATPase that shares similarity with bacterial RuvB helicase (26) and is essential for cell proliferation (26, 27). While its overexpression is linked to several cancers (28–31), evidence also suggests its role in controlling cancer cell proliferation under regulated conditions (32, 33). Surprisingly, silencing the expression of RUVBL-1 affected the translocation of *Ta*PHB-1 in the host cytosol, whose mechanism is only speculative. Considering that RUVBL-1 has chaperone activity (34), one possibility is that the secreted *Ta*PHB-1 remains stable, thus acting as an effector for cellular transformation. However, when RUVBL-1 is quenched in a dose-dependent manner, a decrease in the burden of parasite marker-*Ta*SP was noted using quantitative gene expression analysis. Our observations suggested that the knockdown of BoRUVBL-1 rendered the parasites metabolically inactive, though RUVBL-1 silencing did not affect the proliferation of the host cells *per se*. Interestingly, the RUVBL-1 was found localized near the parasite membrane, where *Ta*PHB-1 also colocalizes. This observation may likely suggest a role for RUVBL-1 in transporting *Ta*PHB-1 into the host cytoplasm, a hypothesis that awaits further validation. In fact, several studies elucidate a pleiotropic mode of action for RUVBL-1. For example, it interacts with c-Myc and acts as a transcriptional cofactor through ATPase and helicase activities (30). In the nucleus, RUVBL-1 interacts with telomere reverse transcriptase (TERT) and facilitates the maintenance of telomerase assembly (30). In the cytoplasm, RUVBL-1 regulates phosphatidylinositol 3-kinase-related protein kinase (PIKK) functions and is involved in nonsense-mediated mRNA decay (35). However, to date, no study has documented the interaction of RUVBL-1 with PHB-1.

In addition to RUVBL-1, *Ta*HSP70 was another important molecule detected in the interactome that may regulate directly or indirectly the interaction between bovine RUVBL-1 and *Ta*PHB-1. The precise nature of these interactions and their functions needs further investigation.

Taken together, the present study demonstrates that the localization of bovine PHB-1 remains unchanged in *Theileria*-infected leucocytes, while parasite prohibitin (*Ta*PHB-1) localizes to the parasite membrane and is also detected in the host cytoplasm of the infected cell. Further, we demonstrated that *Ta*PHB-1 interacts with bovine RUVBL-1. Interactome analysis suggested a possible association of *Ta*PHB-1 with other proteins implicated in actin cytoskeleton organization, protein folding, mRNA processing, and metabolic processes, likely contributing to cellular transformation. We speculate that the interaction between PHB-1 and RUVBL-1 will not only have implications in the arena of parasite biology but also shall shed some light on cancer progression.

## MATERIALS AND METHODS

### Ethics approval.

The procedures for animal experiments were approved by the Institutional Animal Ethics Committee (IAEC) with approval no. IAEC/2021/NIAB/28/AS and performed in accordance with the guidelines of the Committee for the Purpose of Control and Supervision of Experiments on Animals (CPCSEA).

### Chemicals and reagents.

RPMI 1640 was purchased from Gibco, Life Technologies. Fetal bovine serum was purchased from GE Healthcare. Penicillin-streptomycin and TRIzol reagent were obtained from Invitrogen. The make your own Mate & Plate library system and yeast transformation system 2 were purchased from TaKaRa. Synthetic-defined (SD) broth/agar medium, dropout (DO) supplements –Trp, –Leu, –Trp/–Leu, and –Trp/–Leu/–Ade/–His, X-alpha-Gal (X-α-gal), and aureobasidin A (AbA) were obtained from TaKaRa. Plasmids pcDNA3-CFP and pcDNA3-RFP were purchased from Addgene. Antibodies against His-tag and S-tag were purchased from Qiagen and GenScript, respectively. Antibodies against RUVBL-1 were purchased from Proteintech Group. Antibodies against β-tubulin, prohibitin, and myc-tag were obtained from Santa Cruz Biotechnology. Antibodies of TOMM (Abcam) were a kind gift from Naresh Babu V. Sepuri, University of Hyderabad, Hyderabad, India. Antibodies for RPL7A were purchased from Cell Signaling Technology. Secondary antibodies for microscopy, Alexa Fluor 647-conjugated anti-rabbit IgG, and Alexa Fluor 555-conjugated anti-mouse IgG were purchased from Cell Signaling Technology. Fluorescein isothiocyanate (FITC)-conjugated anti-IgY was obtained from IgY Immunologix Pvt. Ltd. Secondary antibodies for Western blotting, anti-mouse IgG conjugated with horseradish peroxidase (HRP), and anti-rabbit IgG conjugated with HRP were obtained from Pierce. Vectashield antifade mounting medium with DAPI was purchased from Vector Laboratories. Trypsin/LysC was purchased from Promega.

### Parasite and cell culture.

The in-house-established *T. annulata*-infected bovine leucocyte (Ana2014 cells) culture was maintained as described previously (36, 37). Briefly, *T. annulata*-infected bovine leucocytes were cultured in RPMI 1640 (Gibco, Life Technologies) reconstituted with 10% heat-inactivated fetal bovine serum (GE Healthcare) and 100 μg/mL penicillin-streptomycin (Invitrogen). Cells were cultured at 37°C in a 5% CO_2_ atmosphere under humidified conditions. Ana2014 cells were treated with buparvaquone at a concentration of 500 ng/mL for 72 h with drug replacement after 48 h. The BoMac cells, a kind gift from Judy Stabel, were cultured in a similar way as Ana2014 cells. HEK293 cells were cultured in Dulbecco’s modified Eagle’s medium (DMEM) reconstituted with 10% heat-inactivated fetal bovine serum and 100 μg/mL penicillin-streptomycin at 37°C in a humidified 5% CO_2_ incubator.

The bovine PBMC were isolated from healthy cattle blood, and macrophage differentiation from the PBMC was performed as described earlier with modifications (36, 38). Briefly, the isolated PBMC were cultured in RPMI 1640 supplemented with 10% heat-inactivated fetal bovine serum for 4 h. The adhered cells were propagated in the same medium supplemented with 100 ng/mL recombinant bovine granulocyte-macrophage colony-stimulating factor (GM-CSF) (Kingfisher Biotech, USA) for 5 days to undergo macrophage differentiation. The differentiated macrophages were used for localization studies.

### *In silico* analysis of TA04375 (*Ta*PHB-1).

The SignalP 3.0 server was used for searching proteins with signal sequences in the proteome of *T. annulata* (39). The proteins expressed in the macroschizont stage were further filtered (21, 22). Prohibitin protein sequences of *T. annulata*, *Theileria parva*, Plasmodium falciparum, Toxoplasma gondii, Cryptosporidium parvum, Babesia bovis, Bos taurus, and Homo sapiens were downloaded from UniProt KnowledgeBase (UniProtKB). The phylogenetic tree was constructed using the maximum likelihood method with a bootstrap value of 100 in MEGA 5 software (40).

The multiple sequence alignment of prohibitin sequences of *T. annulata* was performed using Jalview (41). The CELLO server was used for predicting the subcellular localization of these prohibitins (42, 43).

### Expression, purification, and generation of polyclonal sera against *Ta*SP.

The coding sequence of TA17315 (*Ta*SP) from 76 bp to 495 bp was amplified by PCR from cDNA of Ana2014 cells using specific primers, TASP-F and TASP-R (Table S1). The *Ta*SP was digested with NdeI and XhoI and cloned into the corresponding sites of pET-28a(+) (Novagen). The recombinant construct was transformed into E. coli Rosetta (DE3) competent cells. The culture was induced with 0.5 mM isopropyl IPTG (1-thio–d-galactopyranoside) and further grown at 37°C for 4 h. The recombinant His-tagged *Ta*SP was purified under native conditions. Briefly, cells were pelleted down and lysed by sonication in lysis buffer (50 mM NaH_2_PO_4_ [pH 8], 300 mM NaCl, 10 mM imidazole), and the supernatant was collected by centrifuging at 8,000 × *g* for 25 min at 4°C. The supernatant was passed through the equilibrated Ni-NTA-agarose affinity resin (Qiagen). The resin was washed with wash buffer (lysis buffer with 40 mM imidazole), and the protein was eluted in elution buffer (lysis buffer with 200 mM imidazole). The eluted protein was dialyzed overnight with dialysis buffer 1 (50 mM sodium phosphate buffer [pH 5.8] with 40 mM NaCl) at 4°C. The dialyzed protein was further purified by ion-exchange chromatography. The dialyzed protein was passed through the equilibrated Q-Sepharose fast flow beads (GE Healthcare). The beads were washed with dialysis buffer 1 with a gradual increase in NaCl concentrations of 40 mM, 60 mM, 80 mM, and 100 mM NaCl. The bound protein was eluted with elution buffer (dialysis buffer 1 with 500 mM NaCl). The eluted protein was dialyzed overnight with dialysis buffer 2 (phosphate-buffered saline [PBS], pH 7.2) at 4°C. The purified protein was concentrated using a 10-kDa centrifugal filter (Amicon) to a concentration of 1 mg/mL. The recombinant *Ta*SP was used for raising polyclonal antibodies in chickens at IgY Immunologix Pvt. Ltd.

### Quantitative real-time PCR.

Total RNA was extracted from Ana2014 cells using NucleoSpin RNA Plus (Macherey-Nagel, Germany) according to the manufacturer’s instructions. The quality of the RNA was assessed using a NanoDrop 1000 spectrophotometer (Thermo Fisher Scientific, Inc., USA). RNA with an A_260/280_ (absorbance ratio) of 2.0 was used for cDNA preparation. The cDNA was prepared from 1 μg of total RNA using a PrimeScript 1st-strand cDNA synthesis kit (TaKaRa, Japan) according to the manufacturer’s instructions. Quantitative real-time RCR (qRT-PCR) was carried out using SYBR premix *Ex Taq* (Tli RNaseH Plus; TaKaRa, Japan) and a CFX96TM real-time PCR system (Bio-Rad, USA). Bovine actin was used as the endogenous control. The primers used are listed in Table S1. The expression levels were calculated with the 2^−ΔΔ^*^CT^* method, normalizing with the threshold cycle (*C_T_*) values of the bovine actin.

### Construction of yeast two-hybrid cDNA library (prey library) of *T. annulata*-infected bovine leucocytes.

The total RNA was extracted from Ana2014 cells using TRIzol reagent (Invitrogen) following the manufacturer’s protocol. A total of 1 μg of total RNA after DNase I (Invitrogen) treatment was used for cDNA library preparation using the make your own Mate & Plate library system (TaKaRa). All the procedures were followed according to the manual provided. Briefly, the first-strand cDNA was synthesized using SMART MMLV reverse transcriptase using CDS III primer and SMART oligonucleotide (Table S1), which resulted in known sequences at both ends of the cDNA. Next, the cDNA was amplified by long-distance PCR amplification using Advantage polymerase mix (TaKaRa). The amplified double-stranded (ds) cDNA was purified using a CHROMA SPIN TE-400 column, which retains and traps the DNA molecules of less than 200 bp size. The ds cDNA after CHROMA SPIN TE-400 column purification was used for library construction by *in vivo* homologous recombination in yeast. The purified ds cDNA and SmaI linearized pGADT7-Rec were cotransformed into the Y187 yeast strain using the Yeastmaker yeast transformation system 2 (TaKaRa). The transformation mix was spread onto the SD/–Leu agar plates and incubated at 30°C for 3 to 4 days. All the transformants were harvested and pooled in a freezing medium (yeast extract-peptone-dextrose-adenine [YPDA]/25% glycerol), aliquoted, and stored at −80°C. Also, the number of independent clones was determined.

### Construction of bait plasmid.

The total RNA was extracted from Ana2014 cells, as mentioned above. The cDNA was synthesized using the SuperScript first-strand synthesis system (Invitrogen) following the manufacturer’s protocol. *Theileria annulata* putative prohibitin (TA04375, GenBank accession no. XM_949999, *Ta*PHB-1) was amplified by PCR using its specific primers: TA04375-F and TA04375-R (Table S1). The PCR product was purified using a PCR purification kit (Macherey-Nagel) and digested with NdeI and BamHI restriction enzymes. The digested *Ta*PHB-1 gene was ligated with pGBKT7 (NdeI and BamHI digested) vector plasmid. The recombinant pGBKT7- *Ta*PHB-1 was confirmed by sequencing.

### Expression of bait protein in Y2HGold yeast cells.

The recombinant pGBKT7-*Ta*PHB-1 was transformed into a Y2HGold yeast strain following the Yeastmaker yeast transformation system 2 (TaKaRa). The transformants were screened on an SD/Trp agar plate. A colony from the SD/Trp was inoculated in 5 mL of SD/–Trp broth and cultured overnight at 30°C and 250 rpm. The overnight culture was added to 50 mL of YPDA and cultured at 30°C until the optical density at 600 nm (OD_600_) reached 0.4 to 0.6. The protein extract was prepared by lysing the yeast cells in a cracking buffer, as described previously (44). The protein lysate was resolved on 12% SDS-polyacrylamide gel and electro-transferred onto a polyvinylidene fluoride (PVDF) membrane. After being blocked in blocking solution (3% skimmed milk powder in Tris-buffered saline with Tween 20 [TBST]), the membrane was incubated with myc antibody (Santa Cruz Biotechnology). The expression of myc-tagged *Ta*PHB-1 was detected using horseradish peroxidase (HRP)-conjugated anti-mouse secondary antibody (Pierce) with SuperSignal West Pico chemiluminescent substrate (Thermo Scientific) in a G:BOX Chemi imaging system (Syngene).

### Examining the autoactivation and toxicity of the bait protein in the yeast cells.

The Matchmaker gold yeast two-hybrid system (TaKaRa) protocols were followed to verify the autoactivation of reporter genes by *Ta*PHB-1. Briefly, the recombinant pGBKT7-*Ta*PHB-1 plasmid was transformed into the Y2HGold yeast strain, as mentioned above. The transformation mix was spread on SD/–Trp, SD/–Trp/X-α-Gal, and SD/–Trp/X-α-Gal/AbA agar plates and incubated at 30°C for 3 to 5 days.

### Yeast two-hybrid library screening.

The yeast-two-hybrid library screening was performed according to the protocols of the Matchmaker gold yeast two-hybrid system (TaKaRa). Briefly, a single colony of Y2HGold-pGBKT7-*Ta*PHB-1 was inoculated in 50 mL of SD/–Trp broth medium and cultured overnight at 30°C and 250 rpm. After an OD_600_ of ~0.8 reached, the yeast cells were pelleted down at 1,000 × g for 5 min and resuspended in fresh SD/–Trp broth medium so that the cell density reached 1 × 10^8^ cells/mL. The concentrated culture of the bait was mixed with 1 mL of prey library culture and incubated at 30°C and 40 rpm. After 24 h, the yeast cells were pelleted down at 1,000 × *g* for 10 min and resuspended in 10 mL of 0.5× YPDA/kanamycin broth medium, and the cells were spread onto 150 mm DDO/X/A agar plates. Also, spreading on SD/–Trp, SD/–Leu, and SD/–Leu/–Trp (DDO) agar plates was done to determine the mating efficiency. The agar plates were incubated at 30°C for 3 to 5 days. All the blue colonies which grew on DDO/X/A agar plates were patched on an QDO/X/A agar plate and incubated at 30°C for 3 to 5 days. The plasmids were extracted from all the colonies which grew on the QDO/X/A agar plate using the Easy yeast plasmid isolation kit (TaKaRa). PCR was carried out using the extracted plasmids as templates and using the forward primer (5′-TAATACGACTCACTATAG-3′) and reverse primer (5′-CTGTGCATCGTGCACCATCT-3′) specific to the pGADT7Rec vector plasmid to identify similar size inserts.

### Confirmation of positive interactions and sequencing of the interacting partner of TA04375.

The positive interactions were confirmed by cotransformation of bait and prey plasmids into the Y2HGold yeast strain. After removing the duplicate prey plasmids based on size by PCR, each prey plasmid was transformed into E. coli DH5α competent cells (Invitrogen) and spread on LB/ampicillin agar plates. The plasmids were extracted from the transformants using the NucleoSpin plasmid purification kit (Macherey-Nagel). Each of these prey plasmids and previously constructed bait plasmids was cotransformed into the Y2HGold yeast strain using the Yeastmaker yeast transformation system 2 (TaKaRa). The transformation mix was spread on DDO/X and QDO/X/A agar plates and incubated at 30°C for 3 to 5 days. The plasmids of genuine positive interactions were sequenced. The prey plasmid was sequenced using the primers which were used for PCR. The sequence obtained was analyzed using the BLAST tool of NCBI.

### *Ta*PHB-1-bovine RUVBL-1 interaction study.

The coding sequence of *Ta*PHB-1 was PCR amplified from Ana2014 cell cDNA using its specific primers (Table S1). The *Ta*PHB-1 amplicons were digested with BamHI and SalI and cloned into the corresponding sites on MCS1 of the pETDuet-1 (Novagen) vector plasmid. Also, the coding sequence of *B. taurus* RUVBL-1 was PCR amplified using Ana2014 cell cDNA and its specific primers (Table S1). The amplified RUVBL-1 was digested with NdeI and BglII and cloned into the corresponding sites on MCS2 of pETDuet-1-*Ta*PHB-1.

The recombinant constructs (pETDuet-1-*Ta*PHB-1 and pETDuet-1-*Ta*PHB-1-RUVBL-1 were separately transformed into E. coli Lemo21(DE3) (NEB) chemical competent cells. The cultures were induced with 0.4 mM IPTG and 1 mM rhamnose and further grown at 37°C for 4 h for the expression of recombinant proteins. The cells were pelleted down and lysed by sonication in lysis buffer (50 mM NaH_2_PO_4_ [pH 8], 300 mM NaCl, 10 mM imidazole). The cell lysate was incubated with Ni-NTA-agarose (GE Healthcare) affinity resin overnight at 4°C. The resin was washed with 20 mM imidazole-containing lysis buffer, and finally the His-tagged *Ta*PHB-1 was eluted with 500 mM imidazole-containing lysis buffer. The Western blot analysis of the samples was performed as mentioned in the previous sections. The membrane was incubated with antibodies for His-tag-HRP or S-tag. The S-tagged bovine RUVBL-1 was probed with an anti-rabbit-HRP secondary antibody.

### Colocalization studies.

The full-length *Ta*PHB-1 was PCR amplified from Ana2014 cell cDNA using the specific primers (Sheet S1). The amplified *Ta*PHB-1 was cloned into the pcDNA3-RFP plasmid (no. 13032, Addgene) using the HindIII and NotI restriction sites. Also, the full-length bovine RUVBL-1 was PCR amplified from Ana2014 cell cDNA using the specific primers (Table S1). The amplified RUVBL-1 was cloned into the pcDNA3-CFP plasmid (no. 13030, Addgene) using the HindIII and NotI restriction sites. Both the clones were confirmed by double digestion and sequencing. The recombinant constructs were transfected into HEK293 cells either individually or together using Lipofectamine 3000 reagent (Invitrogen) following the instructions provided. After 24 h of transfection, the fluorescence images of the HEK293 cells were captured using an Axio Observer 7 microscope Apotome 2 (Carl Zeiss).

### Cloning, expression, and purification of recombinant *Ta*PHB-1 and raising of polyclonal antibodies.

The coding sequence of *Ta*PHB-1 was PCR amplified from Ana2014 cell cDNA using specific primers (Table S1). The full-length TA04375 was digested with NdeI and NotI and cloned into the corresponding sites of pET-28a(+) (Invitrogen). The recombinant construct was transformed into E. coli Lemo21(DE3) competent cells (NEB). The culture was induced with 0.4 mM IPTG and 1 mM rhamnose and further grown at 37°C for 4 h. The recombinant His-tagged *Ta*PHB-1 was purified in a denatured condition. Briefly, cells were pelleted down and lysed by sonication in lysis buffer (10 mM Tris-HCl [pH 8], 10 mM EDTA, 100 mM NaCl, 10 mM DTT), and the inclusion bodies were pelleted down by centrifuging them at 13,800 × *g* for 25 min at 4°C. The inclusion bodies were washed with wash buffer 1 (50 mM phosphate buffer [pH 7.2], 10 mM EDTA, 200 mM NaCl, 2 M urea, 1% Triton X-100) and subsequently with wash buffer 2 (50 mM phosphate buffer [pH 7.2], 1 M NaCl). The inclusion bodies were solubilized in solubilization buffer (10 mM Tris-HCl [pH 8], 100 mM NaH_2_PO_4_, 100 mM NaCl, 8 M urea) and incubated with equilibrated Ni-NTA-agarose affinity resin for 2 h at room temperature. The resin was washed with solubilization buffer (pH 6.3). Recombinant *Ta*PHB-1 was eluted in the solubilization buffer (pH 4.3). The eluted pure recombinant *Ta*PHB-1 was used for raising polyclonal antibodies in mice, as described previously (45).

### Affinity purification of *Ta*PHB-1 mouse polyclonal antibodies.

The antibodies specific to *Ta*PHB-1 were purified from mouse sera by immunoblotting as described previously (46). Briefly, 500 μg of the purified *Ta*PHB-1 protein was separated on SDS-PAGE gel and transferred onto PVDF membrane. The membrane was blocked with blocking buffer (5% bovine serum albumin [BSA] in TBST) for 2 h at room temperature. The protein band was stained by Ponceau S staining; the protein band was cut and washed with TBST until the membrane was destained. The membrane strip was incubated with 500 μL of mice serum overnight at 4°C. The strip was washed thrice with TBST. Finally, the bound antibodies were eluted in 2 M glycine (pH 2.5), and the eluate was immediately neutralized with 1 M Tris-HCl buffer (pH 9.0).

### Coimmunoprecipitation.

Coimmunoprecipitation (co-IP) was carried out using a Pierce direct magnetic IP/co-IP kit (Thermo Scientific) according to the instructions provided by the manufacturer. Briefly, 10 μg of affinity-purified *Ta*PHB-1 antibodies were cross-linked to the beads using disuccinimidyl suberate (DSS). Ana2014 cells (40 × 10^6^ cells) were lysed in 1 mL of lysis buffer and incubated with the antibody cross-linked beads overnight at 4°C. The beads were collected on a magnetic stand and washed thrice with the wash buffer. The bound proteins were eluted in the elution buffer, and the eluate was neutralized with the neutralization buffer. The eluate was analyzed for the presence of both *Ta*PHB-1 and bovine RUVBL-1 proteins by Western blot analysis.

### Mass spectrometry analysis of co-IP-eluted proteins.

The co-IP eluate was processed for LC-MS/MS analysis using the in-solution trypsin digestion method. The eluate was subjected to reduction with 20 mM DTT at 56°C for 1 h. Next, alkylation was carried out with 20 mM iodoacetamide (IAA) for 1 h at room temperature in the dark. The proteins were digested with trypsin/LysC (1:30 wt/wt) overnight at 37°C. The digestion reaction was stopped by adding 0.1% trifluoroacetic acid. The digested peptides were purified with C_18_ spin columns. The purified peptides were concentrated using a vacuum evaporator and finally resuspended in 0.1% formic acid. The peptides were analyzed with a Q Exactive HF-Orbitrap mass spectrometer (Thermo Fisher Scientific) coupled with an Ultimate 3000 RSLCnano LC system (Thermo Fisher Scientific). The peptides were injected into a reverse-phase C18 column (PepMap RSLC C18, 2 μm, 100 Å, 75 μm by 50 cm; Thermo Fisher Scientific) and separated by a gradient flow of solvent B (0.1% formic acid in 80/20 acetonitrile/water) from 5% to 90% in 180 min. A parent ion scan was performed with a scan range of 375 to 1,600 *m/z* with a resolution of 60,000. The top 25 intense peaks were fragmented by higher energy collision-induced dissociation (HCD) fragmentation in MS/MS with a resolution of 15,000. The obtained spectra were analyzed using Thermo Proteome Discoverer software version 2.5. The database search was carried out using SEQUEST HT with the following parameters: maximum missed cleavage sites of 2, fragment mass tolerance of 0.02 kDa, and precursor mass tolerance of 10 ppm. Fixed modification, carbamidomethylation of Cys (cysteine), dynamic modification, and oxidation of Met (methionine) were added. The resulting peptides were validated using a percolator at a 5% false-discovery rate (FDR), which validates using posterior error probability (PEP) and *q* value. The interactome analysis was performed using the STRING tool and Cytoscape version 3.8.2 software.

### Immunofluorescence.

The Ana2014 cells were washed twice with PBS and allowed to attach on poly-lysine-coated coverslips. The cells were fixed and permeabilized in 3.7% paraformaldehyde/0.18% Triton X-100 (in PBS) at 37°C for 30 min. The Ana2014 cells were washed once with PBS, and the coverslip was blocked using a blocking buffer (1% BSA in PBS) at 37°C for 1 h. The cells were incubated with the antibodies for RUVBL-1, BoPHB-1, TOMM, *Ta*SP, and affinity-purified antibody *Ta*PHB-1 at 4°C overnight. The cells were washed thrice with PBS and incubated with Alexa Fluor 647-conjugated anti-rabbit IgG (Cell Signaling Technology), Alexa Fluor 555-conjugated anti-mouse IgG (Cell Signaling Technology), and FITC-conjugated anti-IgY (IgY Immunologix) at room temperature for 1 h. The cells were washed thrice with PBS, and the coverslip was mounted on a glass slide with Vectashield antifade mounting medium with DAPI (Vector laboratories). The images were captured using a confocal microscope (Leica SP8, Leica Microsystems) and processed using LAS X software. The images were analyzed with ICY software (47).

### Immuno-electron microscopy.

Ana2014 cells were fixed in 4% paraformaldehyde and 0.2% glutaraldehyde in 0.1 M phosphate buffer (pH 7.4) for 1 h at room temperature. The cells were washed with 0.1 M phosphate buffer. The dehydration and embedding were carried out according to the procedure provided in the low-viscosity embedding kit (Electron Microscopy Sciences). Thin sections of 30 nm were made using an ultramicrotome (Leica UC7). The sections were mounted onto the nickel grid and blocked with 5% BSA in TBS for 20 min at room temperature. The sections were incubated with affinity-purified anti-*Ta*PHB-1 antibodies overnight at 4°C, followed by five washes with TBS. The sections were incubated with gold nanoparticle-conjugated anti-mouse secondary antibodies (Sigma-Aldrich) for 1 h at room temperature, followed by seven washes with distilled water. The sections were stained with uranyl acetate, and images were captured using a transmission electron microscope (JEM 1400, 120 kV, Jeol).

### siRNA transfection.

Ana2014 cells were transfected with either 200 nM control siRNA or 200 nM RUVBL-1 targeting siRNAs (Kaneka Eurogentec S.A.) as described previously (36). The three siRNA sets were used (Table S2). Then, 24 h posttransfection, cells were collected and used to perform qRT-PCR, Western blotting, and immunofluorescence studies as described above.
