# Supplementary material for: Identification of a Novel Interaction between Theileria Prohibitin (TaPHB-1) and Bovine RuvB-Like AAA ATPase 1
Source: Microbiol Spectr. 2023 Jan 18;11(1):e02502-22. doi: 10.1128/spectrum.02502-22 (PMC9927103; doi:10.1128/spectrum.02502-22)
Supplement: Supplemental file 3 — Supplemental material. Download spectrum.02502-22-s0003.pdf, PDF file, 0.7 MB [file spectrum.02502-22-s0003.pdf]

Supplementary figures

Fig S1. Schematic representation of analysis of proteome for identification of proteins with potential to be involved in the transformation of the host cell.

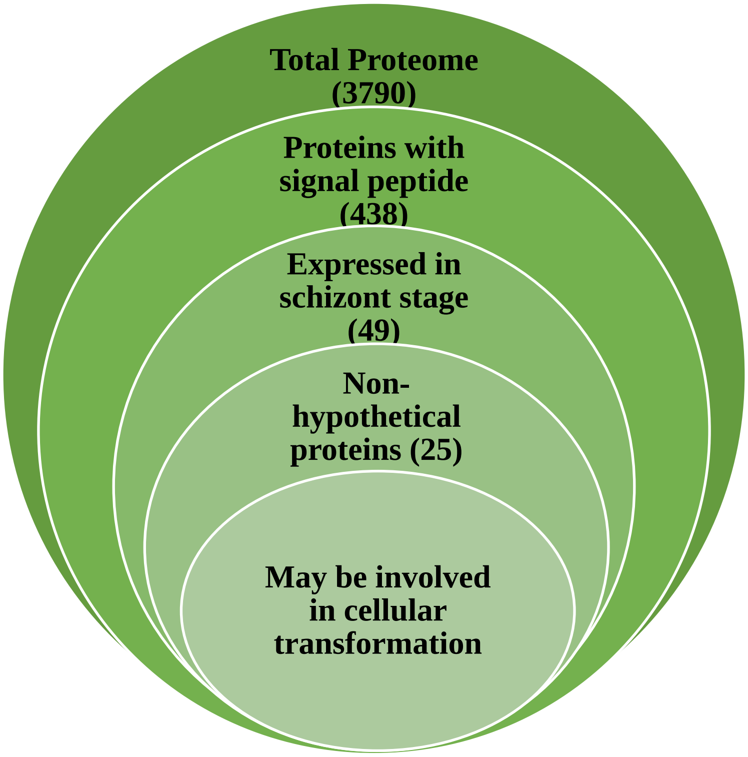

Fig S2. *In silico* analysis of *Theileria annulata* prohibitins. A. Percentage identity matrix of PHB-1 of *T. annulata* (Ta), *T. parva* (Tp), *Babesia bovis* (Bb), *Cryptosporidium parvum* (Cp), *Plasmodium falciparum* (Pf), *Toxoplasma gondii* (Tg), *Bos taurus* (Bt) and *Homo sapiens* (Hs), **B.** Multiple sequence alignment of *Ta*PHBs (Ta04375, Ta19320 and Ta08975) showing SPHF domain with a red color box and the amino acid having 100% identity are shown in color, **C.** Identity matrix of *Ta*PHBs showing that Ta08975 is highly divergent from Ta04375 and Ta19320.

A.

	***Bt*_PHB1**	***Hs*_PHB1**	***Cp*_PHB1**	***Tg*_PHB1**	***Pf*_PHB1**	***Bb*_PHB1**	***Ta*_PHB1**	***Tp*_PHB1**
***Bt*_PHB1**	100.00	100.00	46.13	50.92	46.86	47.60	47.23	46.86
***Hs*_PHB1**		100.00	46.13	50.92	46.86	47.60	47.23	46.86
***Cp*_PHB1**			100.00	59.41	60.37	57.20	60.89	60.52
***Tg*_PHB1**				100.00	65.93	70.74	69.26	68.89
***Pf*_PHB1**					100.00	72.43	71.69	72.06
***Bb*_PHB1**						100.00	79.12	78.39
***Ta*_PHB1**							100.00	98.53
***Tp*_PHB1**								100.00

B.

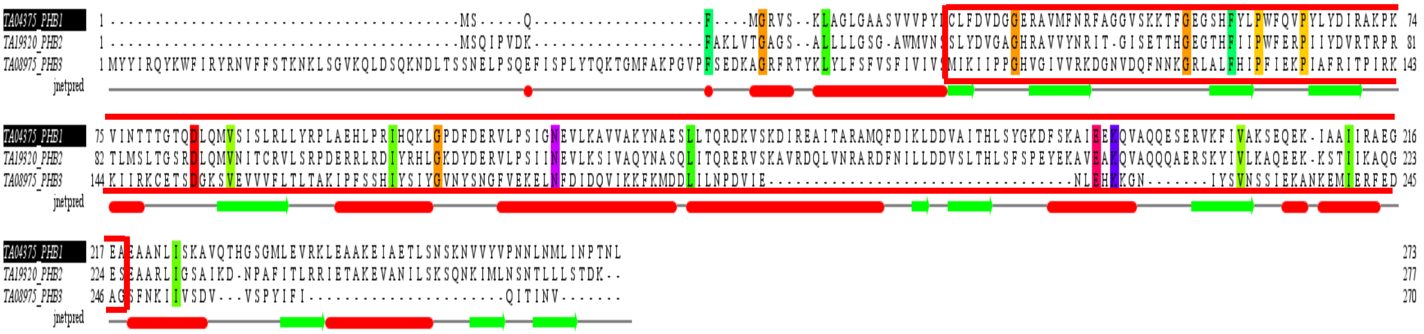

C.

	**TA08975**	**TA04375**	**TA19320**
**TA08975**	**100.00 %**	**18.50 %**	**16.91 %**
**TA04375**		**100.00 %**	**47.76 %**
**TA19320**			**100.00 %**

Fig S3. **Protein expression, purification, chicken polyclonal antibodies of *Ta*SP.** His tagged *Ta*SP protein purification using Ni-NTA agarose beads followed by ion exchange chromatography. M: Marker. 1: Coomassie brilliant blue stained gel- eluate with 500 mM NaCl during ion exchange chromatography, 2: Western blotting of purified *Ta*SP probed with His-tag antibody, 3: Western blotting of Ana2014 cell lysate probed with anti-*Ta*SP IgY antibodies.

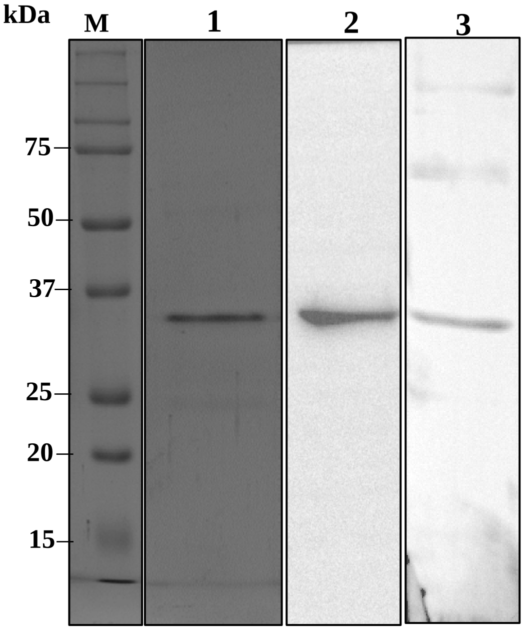

Fig S4. Protein expression, purification, and generation of mice polyclonal antibodies of *Ta*PHB-1. A. His tagged *Ta*PHB-1 protein purification from inclusion bodies under denatured conditions using Ni-NTA agarose beads. M: Marker. 1: Coomassie brilliant blue stained gel-elute with pH-4.3 buffer, 2: Western blotting of purified *Ta*PHB-1 probed with His-tag antibody, 3: Western blotting of purified *Ta*PHB-1 probed with anti-*Ta*PHB-1 mice sera, **B**. **Affinity purification of anti-*Ta*PHB-1 mice sera.** Western blot analysis of Ana2014 and BoMac cells lysates probed with affinity-purified *Ta*PHB-1 polyclonal antibodies. β–tubulin was used as a loading control.

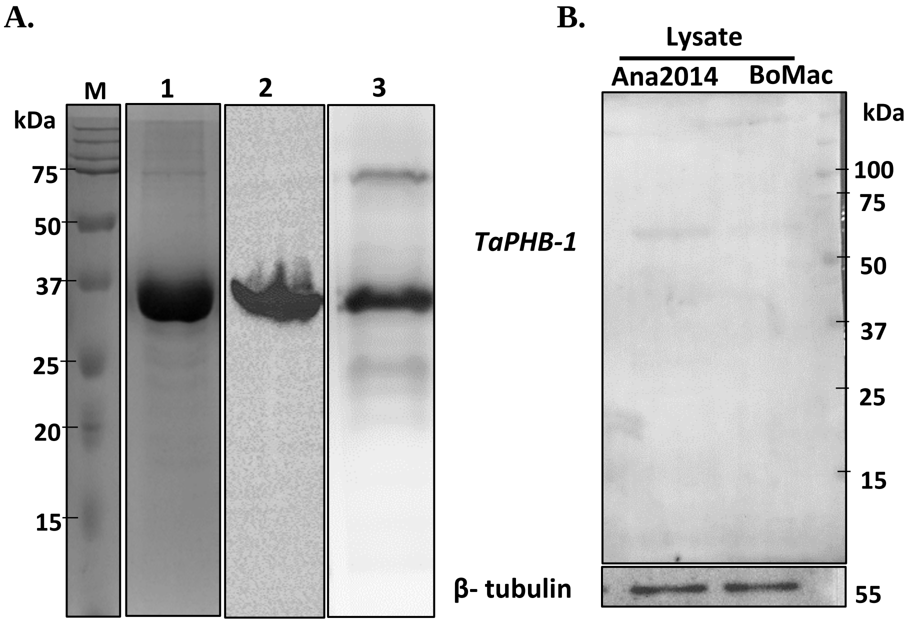

Fig S5. LC-MS/MS analysis of recombinant *Ta*PHB-1. LC-MS/MS analysis of recombinant *Ta*PHB-1 showing 69% coverage and 26 unique peptides, peptides identified by LC-MS/MS are highlighted.

Accession	Description	Coverage [%]	# Peptides	# Unique Peptides
Q4UC35	Prohibitin OS=*Theileria annulata* OX=5874 GN=TA04375 PE=3 SV=1	69	26	26

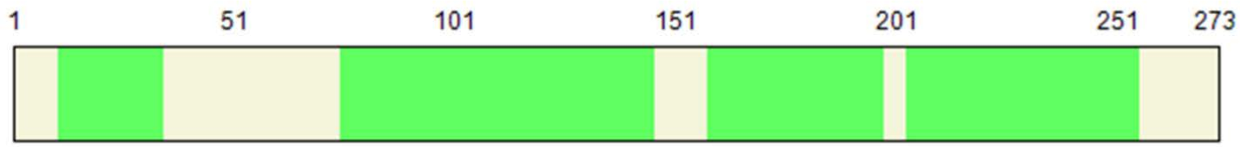


```
     1           11          21          31
                             C
  1  MSQFMGRVSK  LAGLGAASVV  VPYLCLFDVD  GGERAVMFNR
 41  FAGGVSKKTF  GEGSHFYLPW  FQVPYLYDIR  AKPKVINTTT
           O P    P
 81  GTQDLQMVSI  SLRLLYRPLA  EHLPRIHQKL  GPDFDERVLP
                                                O
121  SIGNEVLKAV  VAKYNAESLL  TQRDKVSKDI  REAITARAMQ
161  FDIKLDDVAI  THLSYGKDFS  KAIEEKQVAQ  QESERVKFIV
                                             O
201  AKSEQEKIAA  IIRAEGEAEA  ANLISKAVQT  HGSGMLEVRK
241  LEAAKEIAET  LSNSKNVVYV  PNNLNMLINP  TNL
```

Fig S6. Western blotting of Ana2014 and BoMac cell lysates probed with bovine anti-PHB-1 antibody. Bovine PHB-1 was detected in both samples at the expected molecular weight. β–tubulin was used as a loading control.

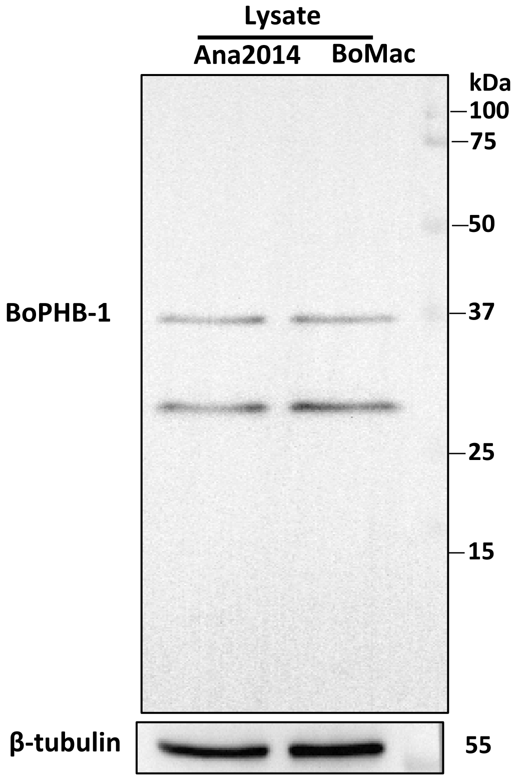

Fig S7. Size distribution of ds cDNA of Ana2014 cells on 1% agarose gel. M: Marker and 1: Ana2014 cells ds cDNA.

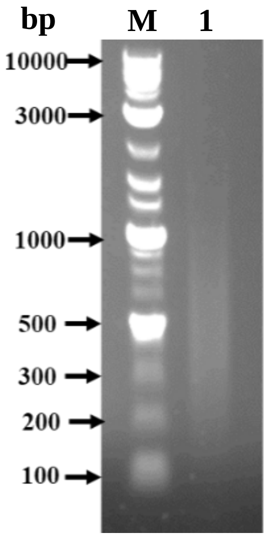

Fig S8. Size distribution of PCR products of prey plasmids using pGADT7-Rec specific primers.

**Size (~bp)**	**Number of prey plasmids**
**350**	**2**
**700**	**3**
**800**	**1**
**900**	**2**
**1000**	**2**
**1100**	**2**
**1200**	**8**
**1500**	**1**
**1400**	**3**

Fig S9. Confirmation of expression induction of *Ta*PHB1 and bovine RUVBL1 in LemoDE3 bacterial cells. Western blotting showing the expression of His tagged *Ta*PHB1 and S-tagged bovine RUVBL1 in LemoDE3 cells. M: Marker, 1: LemoDE3-pETDuet1-*Ta*PHB-1 bacterial cell lysate probed with His-tag antibodies. 2: LemoDE3-pETDuet1- *Ta*PHB-1-RUVBL1 bacterial cell lysate probed with S-tag antibodies.

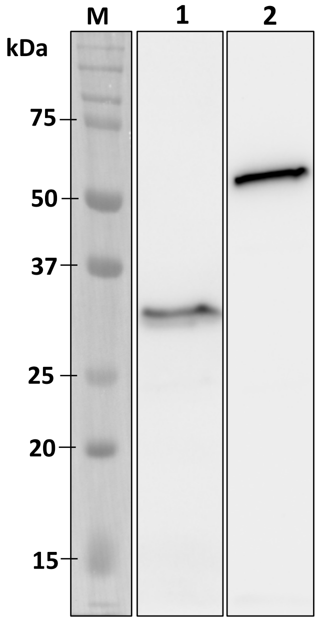

Fig S10. RUVBL1 is not transported to the parasite in Ana2014 cells. A. RUVBL1 was not observed in the parasite but only in the host cell nucleus and cytoplasm, **B.** Quantitative real-time PCR data showing no significant decrease in the transcripts of bovine RUVBL1 after buparvaquone treatment, **C**. The cellular distribution of bovine RUVBL1 is not affected when intracellular parasite is elimitaed by BPQ treatment. Immunostaining of Ana2014 following BPQ treatment with mouse anti-*Ta*PHB-1 antibodies and rabbit anti-Bo-RUVBL1 antibodies. The immunoreactivity was revealed with anti-mouse secondary antibody conjugated to Alexa fluor 555 and anti-rabbit secondary antibody conjugated to Alexa fluor 647. The parasite and host cell nuclei were stained with DAPI. Scale bar- 5 µm, **D.** The IFA images showing no effect in the localization of BoPHB-1 in Ana2014 cells upon silencing the Bo-RUVBL1. Control siRNA was used a negative control. Scale bar- 5 µm.

A.

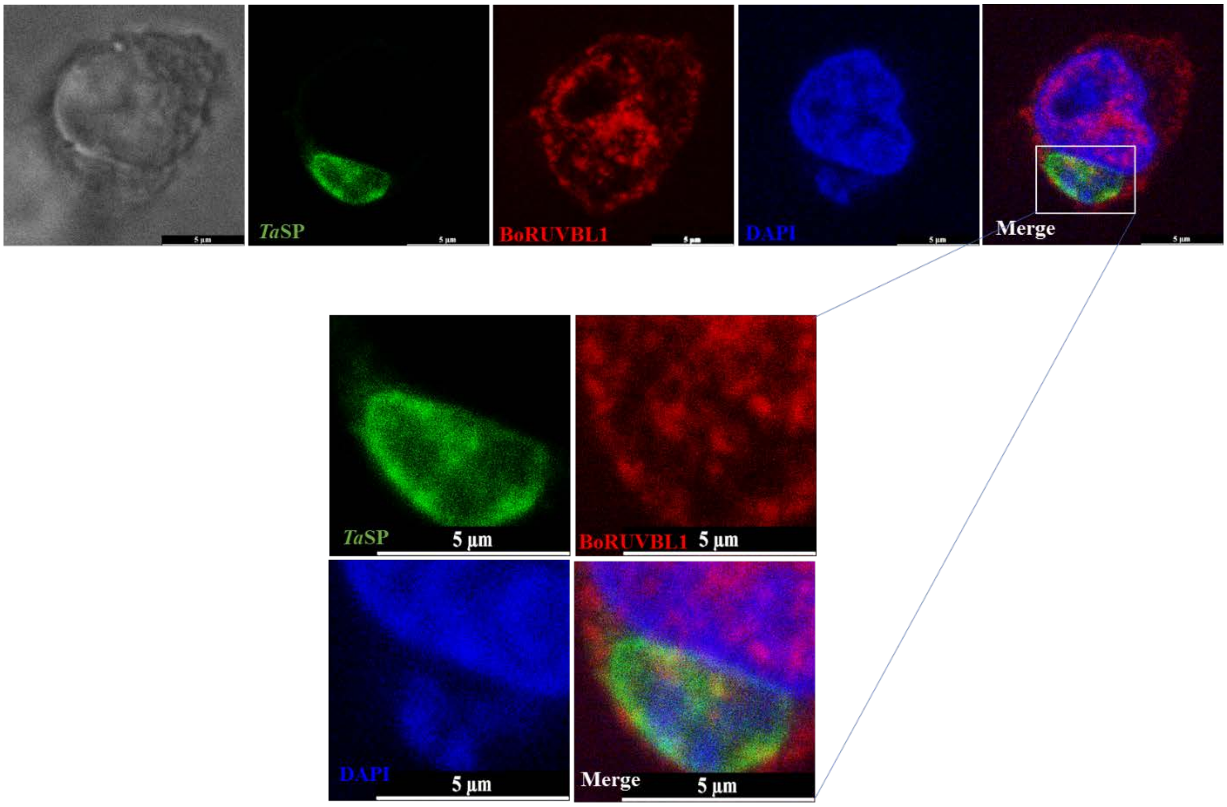

B.

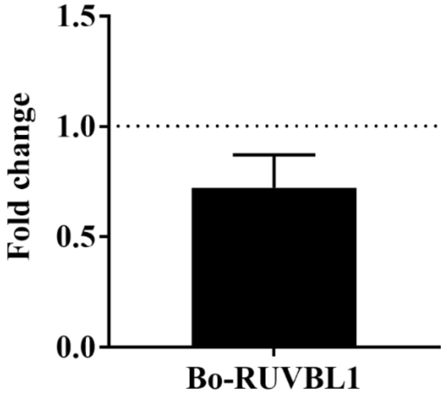
1.5
1.0
0.5
0.0
Fold change
Bo-RUVBL1

C.

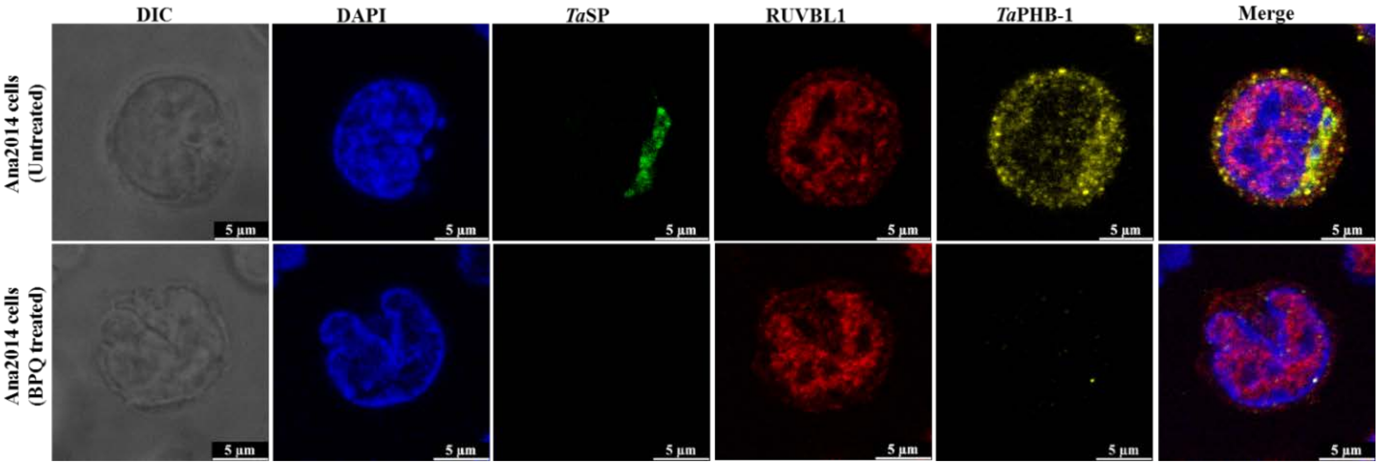
DIC
DAPI
*Ta*SP
RUVBL1
*Ta*PHB-1
Merge
Ana2014 cells (Untreated)
Ana2014 cells (BPQ treated)
5 µm

D.

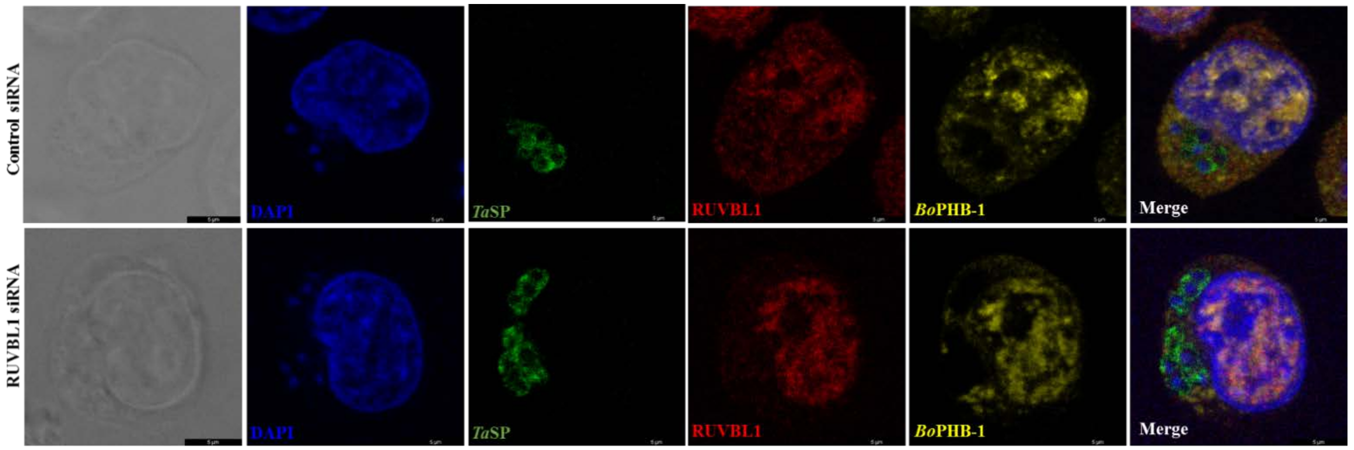
Control siRNA
RUVBL1 siRNA
DAPI
*Ta*SP
RUVBL1
*Bo*PHB-1
Merge

Supplementary tables

Table S1. List of primers used in this study.

Restriction sites are underlined.

S. No.	Name	NCBI reference sequence ID	Sequence (5’ to 3’)
1	CDS III primer	--	ATTCTAGAGGCCGAGGCGGCCGACATG-d(T)30VN where V= A, G, or C and N= A, G, C, or T)
2	SMART oligo	--	AAGCAGTGGTATCAACGCAGAGTGGCCATTATG GCCGGG
3	Ta04375-F	XM_949999.1	gacCATATGTCCCAGTTTATGGGCCG
4	Ta04375-R	XM_949999.1	gcaGGATCCTTAGAGATTAGTGGGATTAATGAG
5	TaSP-F	XM_947650.1	ACTGCATATG GATCGACAACTTAATCCTATC
6	TaSP-R	XM_947650.1	AGTCCTCGAGCTTATCATGATCGGATTTAGATT
7	*Ta*PHB-F	XM_949999.1	gcgGGATCCgATGTCCCAGTTTATGGGCC
8	*Ta*PHB-R	XM_949999.1	gcgGTCGACTTAGAGATTAGTGGGATTAATGAGC
9	*Bo* RUVBL1F	NM_001101076.1	gcgCATATGAAGATCGAGGAGGTG
10	*Bo* RUVBL1R	NM_001101076.1	gcgAGATCTcgCTTCATGTACTTGTCCTGC
11	*Ta*PHB-1 pcDNA1F	XM_949999.1	gagAAGCTTgATGTCCCAGTTTATGGGCC
12	*Ta*PHB-1 pcDNA1R	XM_949999.1	gagGCGGCCGCcaGAGATTAGTGGGATTAATGAGCATA
13	bovine RUVBL1 pcDNA1F	NM_001101076.1	gagAAGCTTgATGAAGATCGAGGAGGTGAAGA
14	bovine RUVBL1 pcDNA1R	NM_001101076.1	gagGCGGCCGCcaCTTCATGTACTTGTCCTGCT
15	*Ta*PHB-1 pET28aF	XM_949999.1	gcgCATATGTCCCAGTTTATGGGCCG
16	*Ta*PHB-1 pET28aR	XM_949999.1	gcgGCGGCCGCGAGATTAGTGGGATTAATGAGC
17	Bovine Actin	AY141970.1	Forward primer- GATCTGGCACCACACCTTCTAC
			Reverse primer- AGGCATACAGGGACAGCACA
18	Bovine RUVBL1 (B0-RUVBL1)	NM_001101076.1	Forward primer- GCACCACTAAGACTCAGCGT
			Reverse primer- TAGAGCCGTCTTGCCAGTTC
19	Bovine Prohibitin (BoPHB-1)	NM_001034572.2	Forward primer- TTGGTCTTGCCTTAGCCGTT
			Reverse primer- GGCACATTACGTGGTCGAGA
20		XM_947650.1	Forward primer- GTGCCCAAGACATCGCA

	T. annulata surface protein (*Ta*SP)		Reverse primer- TAATAGCTTTTGCACGGAGGA
21	TA04375 (*Ta*-PHB1)	XM_949999.1	Forward primer- GCAGGAATCGGAGCGTGTTA
			Reverse primer- TTGGAGAGCGTTTCTGCGAT

Table S2. Details of the siRNA used for silencing Bo-RUVBL1.

S. No.	Sequence (sense strand)
1.	5’- GAAGACGGAGAUCACAGAU -3’
2.	5’- UACUCCACGGAGAUCAAGA -3’
3.	5’- CAAGACAUCCUGUCUAUGA -3’
